# Identification of *Bari* Transposons in 23 Sequenced Drosophila Genomes Reveals Novel Structural Variants, MITEs and Horizontal Transfer

**DOI:** 10.1371/journal.pone.0156014

**Published:** 2016-05-23

**Authors:** Antonio Palazzo, Domenica Lovero, Pietro D’Addabbo, Ruggiero Caizzi, René Massimiliano Marsano

**Affiliations:** 1 Dipartimento di Biologia, Università degli Studi di Bari “Aldo Moro” via Orabona 4 70125, Bari, Italy; 2 Istituto di Biomembrane e Bioenergetica, Consiglio Nazionale delle Ricerche, Via Amendola 165/A, 70126, Bari, Italy; University of Poitiers, FRANCE

## Abstract

*Bari* elements are members of the *Tc1-mariner* superfamily of DNA transposons, originally discovered in *Drosophila melanogaster*, and subsequently identified *in silico* in 11 sequenced Drosophila genomes and as experimentally isolated in four non-sequenced Drosophila species. *Bari*-like elements have been also studied for their mobility both *in vivo* and *in vitro*. We analyzed 23 Drosophila genomes and carried out a detailed characterization of the *Bari* elements identified, including those from the heterochromatic *Bari1* cluster in *D*. *melanogaster*. We have annotated 401 copies of *Bari* elements classified either as putatively autonomous or inactive according to the structure of the terminal sequences and the presence of a complete transposase-coding region. Analyses of the integration sites revealed that *Bari* transposase prefers AT-rich sequences in which the TA target is cleaved and duplicated. Furthermore evaluation of transposon’s co-occurrence near the integration sites of *Bari* elements showed a non-random distribution of other transposable elements. We also unveil the existence of a putatively autonomous *Bari1* variant characterized by two identical long Terminal Inverted Repeats, in *D*. *rhopaloa*. In addition, we detected MITEs related to *Bari* transposons in 9 species. Phylogenetic analyses based on transposase gene and the terminal sequences confirmed that *Bari*-like elements are distributed into three subfamilies. A few inconsistencies in *Bari* phylogenetic tree with respect to the Drosophila species tree could be explained by the occurrence of horizontal transfer events as also suggested by the results of dS analyses. This study further clarifies the *Bari* transposon’s evolutionary dynamics and increases our understanding on the *Tc1-mariner* elements’ biology.

## Introduction

Since their identification eukaryotic Transposable Elements (TEs) developed into an important field of genetic and genomics investigation [1]. Nevertheless, recent advances in sequencing technologies offer a unique and somewhat unappreciated opportunity to increase our understanding of several aspects of the TEs biology, e.g. structure, evolution and regulation (see [2] for a review). Besides their detrimental role as an endogenous source of mutations, TEs transposition and accumulation serve as an evolutionary substrate for genes and genomes evolution [3]. Indeed, inactive TEs play a significant role in macroevolution, contributing in chromosomal rearrangements [4] or being recruited to evolve novel functions [5]. In addition, defective elements and ancient relics of autonomous copies are quite informative to trace the evolution of single TEs families.

The *Tc1-mariner* constitutes one out of 17 super-families of the class II transposons [6]. *Tc1-mariner* elements are widespread among all life kingdoms and their diffusion is mainly due to their simple transposition mechanism, and their proposed ability for cross-species diffusion by horizontal transfer mechanisms [7, 8]. The *Bari* family of transposons belongs to the *Tc1-mariner* superfamily and *Bari*-like elements have been identified in several species of the Drosophila genus. Based on their structural and evolutionary features *Bari*-like elements fall into three distinct subfamilies, *Bari1*, *Bari2* and *Bari3* after the founder elements discovered in *D*. *melanogaster*, *D*. *erecta* and *D*. *mojavensis* respectively. The *Bari1* and *Bari3* subfamilies contain autonomous elements able to perform transposition due to the presence of Terminal Inverted Repeats (TIRs) sequences surrounding a central sequence encoding a functional transposase. On the other hand, *Bari2*-type elements are non-autonomous due to the accumulation of inactivating mutations. Besides the functional criterion, *Bari* elements can be classified standing to the structural differences of the terminal sequences. *Bari1*-type elements harbor short TIRs, usually 28 nucleotides long, called Short Inverted Repeats (SIR) while *Bari2* and *Bari3* possess Long terminal Inverted Repeats (LIR), roughly 250 nucleotides long. Interestingly, non-autonomous *Bari1* elements possessing LIRs have been described in *D*. *ananassae*, a species of the melanogaster group, suggesting that the ancestor of the *Bari1* subfamily had LIRs that were lost during *Bari1* evolution [9]. Regardless their length, both SIR- and LIR- containing elements share three highly similar 18 nucleotides long domains, called Direct Repeats (DRs) [9] [10]. DRs are found within the 250 terminal nucleotides at both ends and are responsible for the transposon-transposase interaction, a crucial step in the transposition event [11, 12]. Such interaction has been previously demonstrated for *Bari1* [13] and *Bari3* [14].

An interesting genomic feature of the *Bari1* subfamily is the presence of a heterochromatic array in the *D*. *melanogaster* species. It was known from previous studies that at least two distinct clusters exist in the reference genome of *D*. *melanogaster*. The first cluster maps to the h39 region [15], a cytological band adjacent to the centromere of the second chromosome of *D*. *melanogaster*, the same cytological band where the *Responder* satellite maps [16]. It contains several tens of *Bari1* copies and is interrupted by a MAX [17] insertion. The second cluster contains few copies and had uncertain heterochromatic map location. Heterochromatic *Bari1*-*Bari1* junctions (Right TIR-Left TIR) are characterized by the deletion of the first two nucleotides (CA) in the left terminus of each element. The terminal copies of both clusters have been also previously characterized [18] thus helping in the reconstruction of their origin [18, 19].

Genome-scale comparison studies are an important tool for both understanding the forces that shaped modern forms of transposable elements, and highlight non-mendelian modes of transposons’ transmission [20]. Early investigations on *Bari*-like transposable elements at the genomic level [9, 21] were essentially performed in 12 Drosophila sequenced genomes available at that time [22, 23]. The availability of 11 additional Drosophila genome draft sequences [24–27] together with the availability of new assembly releases [28] and data from re-sequencing projects [29] prompted us to investigate the genomic distribution of the *Bari* transposon family in a wider pool of studied and unexplored genomes.

In this study, we performed an extensive annotation of the *Bari*-like elements in 23 Drosophila genomes analyzed, and uncovered additional structural variability as compared to previous analyses. In addition, we disclosed the presence of MITE-like forms of the *Bari* transposons in 7 Drosophila species including, interestingly, species apparently devoid of full-length *Bari* elements. Analyses of the integration site of *Bari* elements revealed a preference for AT-rich sequences in which the TA target is duplicated upon integration. Furthermore, annotation of unrelated TEs insertions in the proximity of *Bari* elements revealed significant co-occurrence of other *Tc1-mariner* elements while class I TEs avoid these regions. Finally, we propose that incongruences revealed by our phylogenetic analyses could be explained by horizontal transfer events. Taken together our results significantly increase our understanding of the evolution of *Bari* elements.

## Materials and Methods

### Bari transposon search strategy and sequence analyses

Searches for *Bari* homologous elements were carried out in Drosophila species listed in S1 Table. A BLAST strategy was applied to identify of *Bari*-like elements at the NCBI WGS (Whole Genome Shotgun) database (http://www.ncbi.nlm.nih.gov/genbank/wgs/) or at the FlyBase database [30]. Query sequences for tBLASTn searches were either the *Bari1* or *Bari3* transposases (GenBank CAA47913 and conceptual translation of GenBank accession CH933806 position 6274049–6275068 respectively). Queries for BLASTn searches were performed using either the whole DNA sequence or the 250 terminal nucleotides containing the three DR sequences of *Bari1* or *Bari3*. All BLAST analyses were performed using the default parameters. Subject sequences from tBLASTn searches with E < 10^−120^ and similarity greater than 75% over the whole transposase length (339 amino acids), were further analyzed. The threshold E-value was set to higher values in BLASTn searches aimed at MITEs identification (E >10^−20^).

Two criteria were used to identify full-length *Bari*-like elements; 1) the detection of a high-scoring subject sequence by tBLASTn search, using either the *Bari1* or the *Bari3* transposase protein as query sequence; 2) the presence of homologous DRs in the terminal sequences, surrounding the coding region of the elements. Terminal inverted repeats and homologous DRs in the transposon’s termini were identified by a combined analysis using the Dot Plot matrix analyses implemented in the DNA Strider package software [31], the Einverted software (http://embossbioinformaticsnl/cgi-bin/emboss/einverted) and by multiple sequence alignment of terminal ends of *Bari*-like elements as previously described [9].

Elements’ names were assigned according to the binary nomenclature used in Repbase [32, 33], consisting of the subfamily identifier (either *Bari1*, *Bari2 or Bari3*) followed by the species identifier (i.e. the first letter of the genus and three letters of the species name to avoid ambiguity for some species). Similarly, MITE’s elements names look like Bari_Dxyz_MITE-# (where “xyz” is a three-letters species identifier) followed by a number (#) to distinguish different MITEs subfamilies, where necessary.

Once a novel full-length element was identified, it was used to identify other full-length copies as well as truncated elements, by BLASTn analyses against species-specific WGS database. Each copy was then carefully annotated (S2 Table). Additional BLASTn searches were performed using as query the *mel-ER* (consensus), the *sim-ER* and the *sec-ER* elements (here named *Bari2_Dmel*, *Bari2_Dsim* and *Bari2_Dsec* in the text) described in [9], three *Bari2*-type elements in the genomes of *D*. *melanogaster*, *D*. *simulans*, and *D*. *sechellia* respectively. Redundancy can be excluded for all elements except for split elements, i.e. sequences overlapping either the beginning or the end of a genomic scaffold in which they have been identified. These sequences have been annotated as “partial” elements in S2 Table and were counted as elements. No further analysis was performed using partial elements. Redundant elements were removed on the basis of the flanking sequences comparison. Global alignments were performed using LALIGN (http://wwwchembnetorg/software/LALIGN_formhtml). The Open Reading Frames (ORFs) were inferred using ORF Finder (http://wwwncbinlmnihgov/gorf/gorfhtml) or using a custom script (available upon request) written in Biopython (version 1.63) [34]. The similarity with previously reported sequences was established using CENSOR [35] at the RepBase database [36].

dS analysis was performed using the Nei and Gojobori method [37] implemented in MEGA5 [38]. Fisher’s exact test (one-tailed) was conducted using 2 x 2 tables to verify if transposon dS values were statistically lower than those presented by the host genes.

The number of base substitutions per site between sequence pairs was calculated using MEGA 5.0 [38]. Analyses were performed using the Kimura 2-parameter model. Rate variation among sites was modeled with a gamma distribution (shape parameter = 1). All ambiguous positions were removed for each sequence pair. The whole transposon sequence alignment was used to perform Plotcon analyses (http://emboss.bioinformatics.nl/cgi-bin/emboss/help/plotcon) by moving a window of 100 nucleotides along the aligned sequences. Sequences marked as “partial sequence” in S2 Table were excluded from p-distance and Plotcon analyses. Data analysis was carried out with the R System (R version 3.1.0) (https://www.r-project.org). The non-parametric Kruskal-Wallis test and Tukey test were used to assess significance for the observed differences, considered significant at p values<0.05. Hartigan dip test was carried out to assess unimodality/multimodality of the observed similarity values distributions [39].

### Insertion sites analyses

WebLogo analyses [40] were performed in those species where it was possible to retrieve at least four flanking sequences of equally oriented *Bari* elements. A larger scale insertion site analysis was also performed. Regions of 2500 bp flanking downstream and upstream the insertion sites of *Bari* elements, when available, were analyzed with RepeatMasker-open-4.0.5 (http://www.repeatmasker.org) using the RepBase20.02 dataset. The same analysis was performed, as control, on 1000, non-overlapping, random sequences, 2,5 kb in length, irrespective of their gene content selected from species containing at least four *Bari* insertions, taking care to avoid regions with internal sequencing gaps. The distance of each masked transposon from the sequence origin (fixed at the first base flanking *Bari* elements or to the first nucleotide of the randomly-selected scaffold) was calculated and used to create a positions dataset. The detected distances were then classified in 5 distance ranges from the sequence origin, considering a 500 bp cumulative increment (0–500; 0–1000; 0–1500; 0–2000 and 0–2500). Poisson distribution has been applied to the data, given that the repeat frequency in 1000 random regions was considered as the expected value, and the frequency in the *Bari* flanking regions as the observed value to test. Sequence accessions and coordinates of the control dataset are reported in S1 File. Relative probability was calculated to identify a significant association between *Bari* and the selected repeat families.

### Multiple alignments and phylogenetic analysis

Multiple sequence alignments were performed using ClustalW2 [41] with default parameters. Nucleotide sequences relative either to the Coding Sequence (CDS) or to the terminal sequences were used in the analyses of *Bari* elements. The *Bari1_Dsim* and *Bari1_Dsec* elements were not included in the multiple alignment of terminal sequences and transposase sequences because they are identical in sequence to the *Bari1_Dmel* element. The *D*. *yakuba* element was excluded from the alignment of TIR sequences because it has been re-classified as MITE, and thus included in the MITE elements’ analysis. The BioEdit software was used for alignment editing and visualization [42]. Alignment slices were obtained using the Alignment Slicer tool (http://www.hiv.lanl.gov/content/sequence/Slice_Align/).

Species phylogeny was based on the alignment of nine concatenated orthologous CDSs encoding subunits of the V-ATPase complex, for which orthologous genes could be identified in the 23 species analyzed. FlyBase annotation symbols, GenBank accession of mRNA and length of *D*. *melanogaster* CDSs used in this analysis are the following: CG3762 NM_143747 (1845 bp), CG2934 NM_130724 (1054 bp), CG7071 NM_142770 (909 bp), CG34131 NM_001043279 (483 bp), CG12403 NM_001273497 (1845 bp), CG1088 NM_169073(681 bp), CG7007 NM_143753 (639 bp), CG3161 NM_057453 (480 bp), CG6213 NM_058089 (354 bp). The jModelTest program v 2.1.7 [43] [44] [45] was used to select the simplest evolutionary model that fitted adequately the sequence data. A Bayesian Markov chain Monte Carlo (MCMC) method implemented in BEAST package 1.8.0 [46, 47] was used for Bayesian analysis. The MCMC chains were run for at least 50 million generations and, sampled every 5.000 steps. Convergence was assessed on the basis of the effective sampling size after a10% burn-in. Phylogenetic trees were visualized and edited using FigTree 1.4.2 software (http://treebioedacuk/software/figtree/). Paris [48] and S [49] elements were used as outgroups.

## Results

### *Bari* elements identification and their structural diversity in 23 sequenced genomes

We applied a BLAST-based search to identify insertions of *Bari*-like elements in 23 Drosophila genomes (S1 Table). The evolutionary time of the species analyzed in this work spans roughly 40 million year [50]. We used the full-length *Bari1* and *Bari3* transposase as query in tBLASTn analyses, and the whole transposon DNA sequences in BLASTn analyses against the genome shotgun database or reference genomes. Using this approach, we identified 401 *Bari*-like elements comprising full-length copies, truncated elements and MITEs. The actual number of *Bari* insertions annotated in the species analyzed could be slightly inflated because we have also annotated partial sequences (53 out of 249 sequences in S2 Table), i.e. split sequences overlapping either the beginning or the end of a genomic scaffold in which they have been identified, for which we cannot exclude redundancy. Redundancy can be instead excluded in the remaining sequences for which we compared the flanking sequences to establish uniqueness in the dataset. Inflation is particularly relevant in *D*. *pseudoobscura* (22/33 total insertions) and in *D*. *miranda* (10/16 total insertions). However, inflation could be compensated by the possible underestimation of the *Bari* copy number due to the draft status of the genome assembly in some species. New *Bari*-related sequences were annotated in 11 species (namely *D*. *biarmipes*, *D*. *bipectinata*, *D*. *rhopaloa*, *D*. *takahashii*, *D*. *kikkawai*, *D*. *eugracilis*, *D*. *ficusphila*, *D elegans*, *D*. *suzukii*, *D*. *miranda*, and *D*. *albomicans*). For clarity purposes, data concerning full-length and truncated elements are presented separately from data concerning MITEs. The features of representative elements (i.e. the top scoring and the most complete subjects after BLAST search) are summarized in Table 1, while information concerning all the annotated insertions can be found in S2 and S3 Tables.

**Table 1:** *Bari* elements features in the analyzed genomes.

Drosophila species	Length(bp)	TIR structure	Representative sequence (GenBank accession)	Copies detected	3DRs -containing sequence (bp)	Left vs Right terminal sequences similarity (%)	TNP (aa)	% sim vs Ba1TNP (%)	%sim vs Ba3TNP (%)	Sub-family	Element’s name
melanogaster	1726	SIR-3DR	X67681	69	254	85.2	339	100	79,7	Bari1	Bari1_Dmel
melanogaster	1730	LIR-3DR	reconstructed	10	255	92.2	334*	74	76	Bari2	Bari2_Dmel
melanogaster	116	SIR-1DR	AE013599.5	4	NA	NA	NA	NA	NA	MITE	Bari_Dmel_MITE1
melanogaster	692	SIR-1DR	JSAE01000428.1	1	NA	NA	NA	NA	NA	MITE	Bari_Dmel_MITE2
simulans	1726	SIR-3DR	NC_011089.1	8	254	85,2	339	99	79	Bari1	Bari1_Dsim
simulans	1222	ND	NT_167066.1	1	ND	ND	310*	61	58	Bari2	Bari2_Dsim
simulans	77	SIR-1DR	AASU01036800.1	1	NA	NA	NA	NA	NA	MITE	Bari_Dsim_MITE
sechellia	1727	SIR-3DR	CH481259	7	254	85,2	339*	97	77	Bari1	Bari1_Dsec
sechellia	1265	ND	NW_001999696.1	1	ND	ND	329*	56	55	Bari2	Bari2_Dsec
sechellia	77	SIR-1DR	AAKO01017469.1	24	NA	NA	NA	NA	NA	MITE	Bari_Dsec_MITE1^@^
sechellia	77	SIR-1DR	AAKO01010824.1	25	NA	NA	NA	NA	NA	MITE	Bari_Dsec_MITE2^@^
yakuba	467	LIR-3DR	AAEU02002077.1	1	NA	NA	NA	NA	NA	MITE	Bari_Dyak_MITE
erecta	1639	LIR-3DR	Y13853	16	254	93,8	325*	84	85	Bari2	Bari2_Dere
ficusphila	687	SIR-1DR	AFFG02006632.1	36	NA	NA	NA	NA	NA	MITE	Bari_Dyak_MITE
eugracilis	1020	ND-3DR	AFPQ02005153	1	230	NA	NA	NA	NA	Bari1	Bari1_Deug
biarmipes	1728	LIR-3DR	AFFD02006349	9	262	93,9	335*	88	77	Bari1	Bari1_Dbia
suzukii#	1689	LIR-3DR	reconstructed	4	255	92,2	333*	82	62	Bari1	Bari1_Dsuz
suzukii	398	SIR-1DR	AWUT01015981.1	11	NA	NA	NA	NA	NA	MITE	Bari_Dsuz_MITE
takahashii§	1825	LIR-3DR	AFFI02005672	2	254	100	373*	74	58	Bari1	Bari1_Dtak
takahashii	78	SIR-1DR	AFFI02008544	8	NA	NA	NA	NA	NA	MITE	Bari_Dtak_MITE
elegans	78	SIR-1DR	AFFF02008066.1	4	NA	NA	NA	NA	NA	MITE	Bari_Dele_MITE
rhopaloa	1726	LIR-3DR	AFPP02028090	7	255	100	339	84	65	Bari1	Bari1_Drho
rhopaloa	78	SIR-1DR	AFPP02033070.1	37	NA	NA	NA	NA	NA	MITE	Bari_Drho_MITE
kikkawai	1720	SIR-3DR	AFFH02005270	6	247	59,5	339*	78	62	Bari1	Bari1_Dkik
ananassae	1727	LIR-3DR	AAPP01019820	16	255	93,7	337*	89	77	Bari1	Bari1_Dana
bipectinata#	1729	LIR-3DR	Reconstructed	2	255	97,6	339*	84	65	Bari1	Bari1_Dbip
pseudoobscura	1706	LIR-3DR	AADE01010081	33	243	78,5	339	81	85	Bari3	Bari3_Dpse
persimilis	1704	LIR-3DR	AAIZ01001634	18	242	80,2	339	81	85	Bari3	Bari3_Dper
miranda	1704	LIR-3DR	AJMI02000744	15	243	64,7	339	67	73	Bari3	Bari3_Dmir
willistoni	1725	LIR-3DR	AAQB01008459	6	254	95,7	339	79	78	Bari3	Bari3_Dwil
mojavensis	1717	LIR-3DR	AAPU01011127	15	256	99,6	339	80	100	Bari3	Bari3_Dmoj
virilis	1645	LIR-3DR	AANI01016007	3	255	56,5	322*	78,2	78,2	Bari2	Bari2_Dvir
albomicans£	109	ND-ND	ACVV01103928	1	NA$	NA	ND	NA	NA	Bari2	Bari2_Dalb
grimshawi	ND	ND	ND	ND	ND	ND	ND	ND	ND	ND	NA

GenBank accessions are provided (column 4) for sequences representing the *Bari* elements identified in a given species. Left vs Right terminal sequences similarity (column 7) refers to the DRs-containing sequences (roughly 250 bp).

Symbols legend

*: inferred from a reconstructed transposase gene

§: a MITE nested insertion was removed

#: element reconstructed from two different truncated elements

£: sequence identified by BLASTn analysis

$: sequence containing two DRs

@: Described by Dias and Carareto [21].

ND (NA): none detected (not applicable).

**Fig 1:**
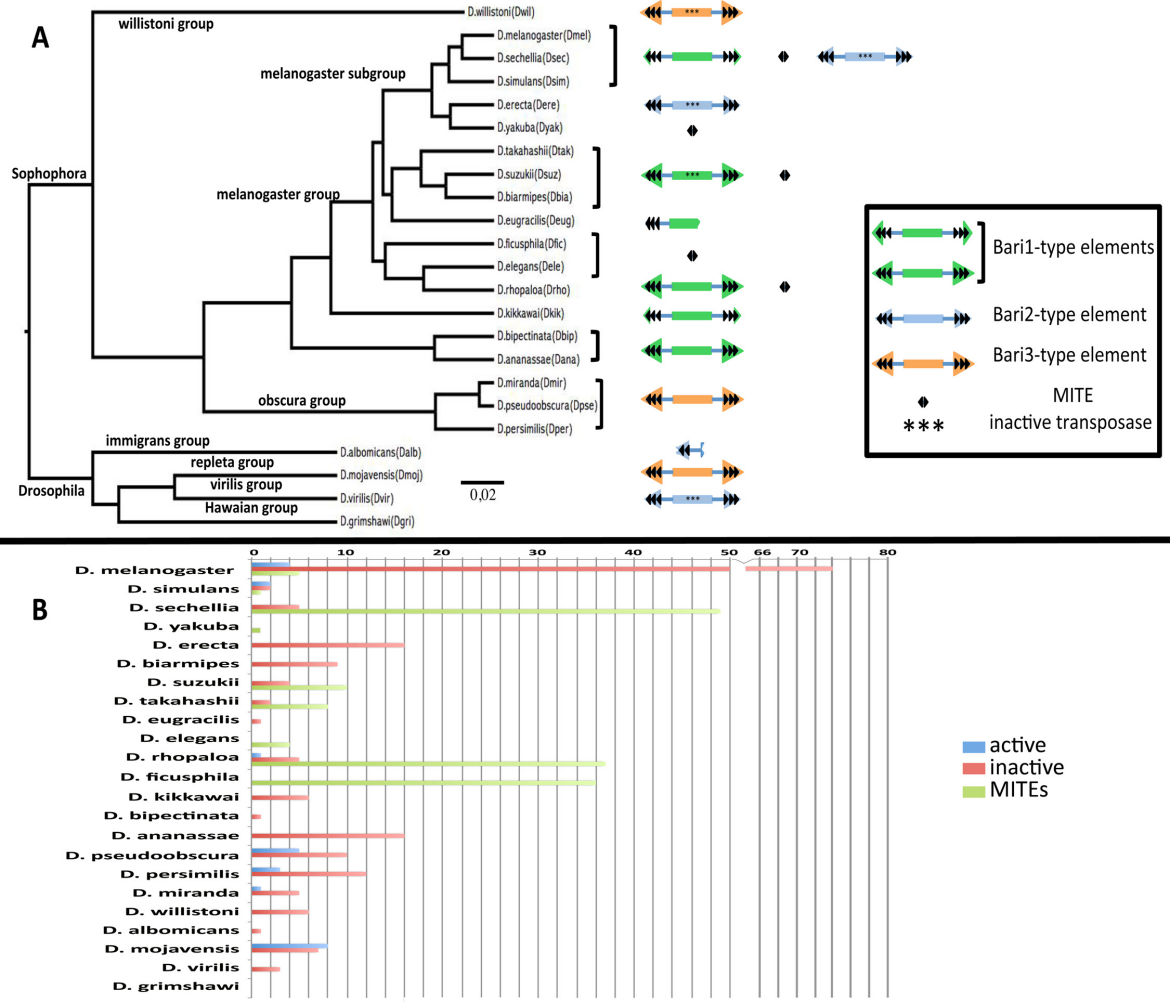
Overview of the distribution and structure of Bari elements in the species analyzed. (A). Bayesian phylogenetic tree (GTR+G+I model) of the species analyzed with a schematic structure of the *Bari* elements identified in each species (not in scale). All nodes are highly supported (posterior probability >0,9). Colored triangles represent subfamily-specific TIRs (see graphical boxed legend). DRs are depicted as black arrowheads. In species containing only truncated elements, elements are depicted using broken boxes. Species harboring only inactive elements due to mutations in CDS are marked with “***”. (B). Histograms recapitulating number and type of *Bari* elements in each analyzed species (see also S2 and S3 Tables).

A snapshot of the distribution of *Bari* subfamilies and the presence of potentially active elements in the species analyzed is reported in Fig 1. Details relative to individual insertions identified in this study are reported in S2 and S3 Tables. The presence of *Bari*-like elements in every genome analyzed, with the exception of *D*. *grimshawi*, confirms previous evidence on the widespread diffusion of this transposon family in the Drosophila genus. As can be observed in Fig 1A, distinct *Bari* subfamilies have colonized specific genomes, with little subfamilies overlaps, as can be observed in the species of the melanogaster complex (*D*. *melanogaster*, *D*. *simulans*, *D*. *sechellia*) for the *Bari1* and *Bari2* subfamilies.

*Drosophila melanogaster* contains the highest number of *Bari*-like element copies (84 elements annotated) mainly clustered in a specific heterochromatic region, (best described in the next paragraph), followed by *D*. *pseudoobscura* (33 insertions detected), while *D*. *albomicans* (1 element) virtually lacks *Bari* elements and *D*. *grimshawii* is devoid of *Bari* elements. A preliminary analysis of all the elements identified suggests that *Bari*-like elements have variable length ranging from 17 bp (S2 Table, *D*. *melanogaster* sheet, element #25) to 4353 bp (S2 Table, *D*. *ananassae* sheet, element #12), due to the existence of canonical copies, truncated copies (i.e. transposon chunks and elements overlapping the extremities of the sequence contigs) or other mobile elements nested within *Bari* elements. The median length of all annotated *Bari* elements is 696 bp (IQR = 1635 bp; max. length = 4353; min. length = 17). In more details, the median length of full-length and truncated elements (S2 Table) is 1704 bp (IQR = 593; max. length = 4353; min. length = 17), while the median length of MITEs (S3 Table) is 82 bp (IQR = 394; max. length = 939; min. length = 69). Considering only those elements containing two terminal sequences (each one containing three DRs) framing an intervening sequence, irrespective of its coding potential (134 elements) *Bari*-like elements look more homogeneous (median length = 1723 bp; IQR =; min. length = 746 bp in *D*. *pseudoobscura*; max length = 4353 bp in *D*. *ananassae*). The TA dinucleotide TSD, a feature of the *Tc1-mariner* superfamily, was identified in 60 out of these 134 sequences. Twenty-four copies of *Bari*, distributed in seven species (*D*. *melanogaster*, *D*. *simulans*, *D*. *rhopaloa*, *D*. *persimilis*, *D*. *pseudoobscura*, *D*. *miranda* and *D*. *mojavensis*) can be considered as autonomous elements due to the presence of two terminal sequences (either with LIR or SIR structure) bracketing a CDS encoding homologous *Bari* transposase.

**Fig 2:**
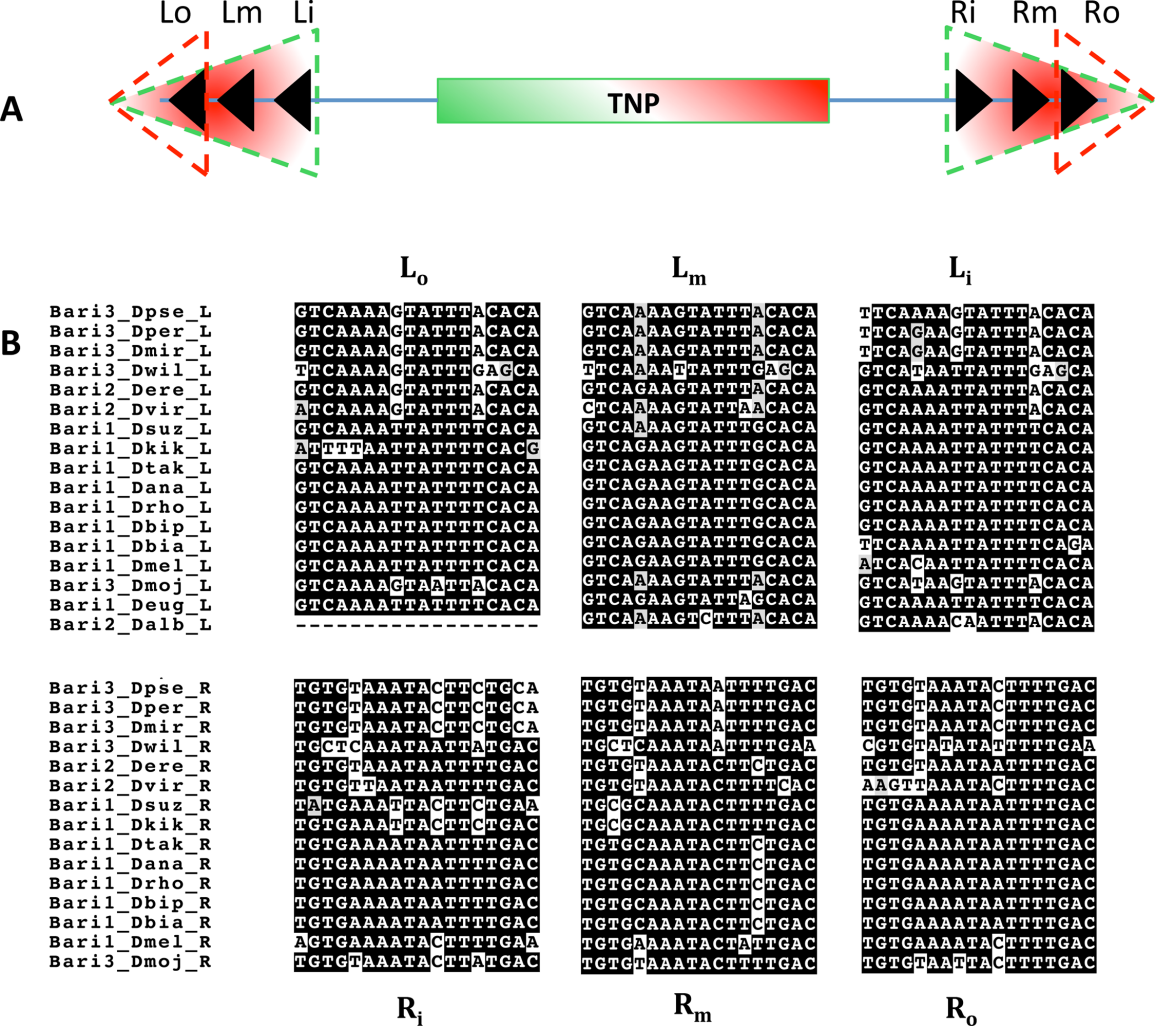
The terminal sequences of *Bari* elements. (A) Schematic representation recapitulating the structure of *Bari* elements. Black arrowheads represent DRs within either the LIRs (green dashed triangles) or SIRs (red dashed triangles) framing a central transposase gene (TNP). See the main text for detailed description of elements belonging to single subfamilies. (B) Alignment slices relative to the conserved blocks of 18 nucleotides (DRs) in the left (Lo, Lm, Li) and right (Ro, Rm, Ri) termini of *Bari*-like transposons. Note that *Bari1_Dmel*, *Bari2_Dere* and *Bari3_Dmoj* correspond to the previously described *Bari1*, *Bari2* and *Bari3* respectively. This analysis was performed using the representative sequences shown in Table 1. Lo, Left outer; Lm, Left middle; Li, Left inner; Ro, Right outer; Rm, Right middle; Ri, Right inner.

**Fig 3:**
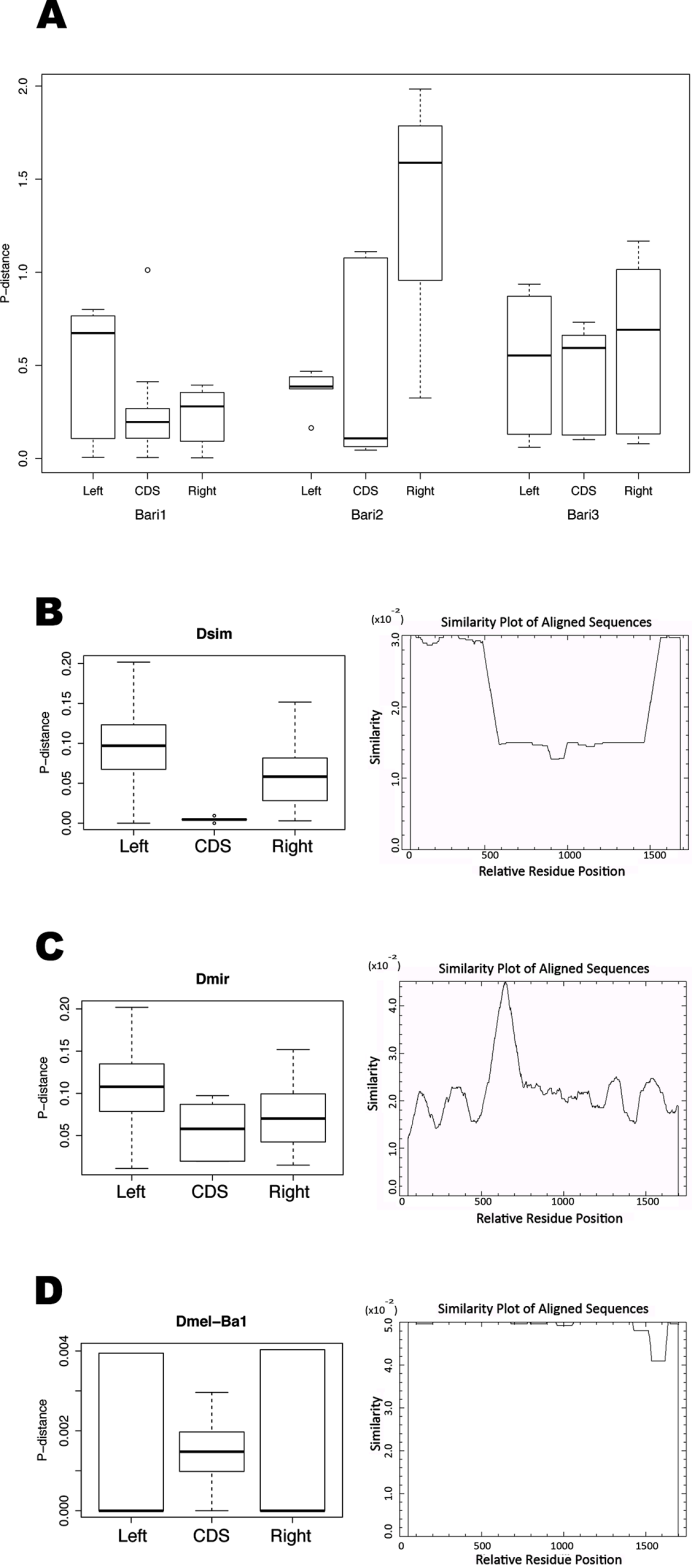
Number of base substitutions per site (p-distance) and graphic representation of the deterioration profiles for *Bari* elements. Bottom and top of the boxes represent the first and third quartiles, the line inside each box represent the median value; whiskers mark the data within the 1.5 IQR range. Outliers are depicted as dots outside the whiskers. (A) Inter-species analysis of the three *Bari* subfamilies. Boxplots report the p-distance relative to the 5’ (Left) and 3’ (Right) sequences containing the DRs (roughly 250 nucleotides analyzed) and to the region homologous to the transposase gene (CDS). Panels B, C, D report examples of intra-species analyses. (B) An example of *Bari* elements showing a significant divergence of both TIRs if compared to the CDS. (C) An example of *Bari* elements showing a significant divergence of a single TIR if compared to the CDS. (D) An example of *Bari* elements not showing a significant TIRs divergence compared to the CDS. The number of sequences used to perform single analyses is reported in S1 Fig. Statistical significance for each analysis is shown in S4 Table.

The presence of three conserved DR sequences within the 250 terminal nucleotides bracketing the transposase gene, typical of the *Bari* family [9], suggest that all the new elements identified are *bona fide Bari*-like elements (Fig 2). We performed a comparative analysis to estimate the sequence variability of *Bari* elements and the deterioration profile both at the inter-species and intra-species level. We calculated the pairwise distance (p-distance) relative to the left and right terminal sequences, comprising the DRs, and to the transposase coding sequence. At the inter-species level, no significant differences can be observed among the CDS and the terminal sequences in the three *Bari* subfamilies (Fig 3A and S4 Table), suggesting the uniform distribution of point mutations in the three regions analyzed. However, some differences were observed by analyzing the same elements at the intra-species level, where the p-distance analysis was also coupled with the determination of the degradation level of the *Bari* elements in each species. As an example, three representative results obtained are shown in Fig 3 (see S1 Fig for complete results). In two species (*D*. *willistoni* and *D*. *simulans*, Fig 3A) the p-distances at the terminal sequences are significantly different if compared to the central region (S4 Table), suggesting an increased mutational load at the terminal sequence level. In four species one of the TIRs has a significantly higher distance if compared to the CDS (*D*. *ananassae D*. *erecta*, *D*. *pseudoobscura*, *D*. *miranda*, Fig 3B). In the remaining three species (*D*. *melanogaster*, *D*. *persimilis*, *D*. *mojavensis*, Fig 3C) no significant difference was observed, suggesting uniform mutation pattern. The respective degradation profiles show that truncated elements are generated in each of the analyzed species by deletion and/or unrelated TEs nested insertions, occurring without any obvious positional preference pattern.

A description of *Bari* elements in those genomes containing few copies, and thus not analyzed as described above, is given below.

The genome of *D*. *takahashii* contains two inactive elements due to insertions and deletions. The *Bari1_Dtak* element was reconstructed removing an unreported MITE insertion (not shown). *Bari1_Dtak* includes a 912 bp-long ORF encoding a protein that shares 81% and 72% similarity with *Bari1* and *Bari3* transposase respectively framed by two LIRs containing 3 DRs.

The *Bari1*_*D*.*rho* element coding sequence encodes a protein 84% similar to the *Bari1* transposase, framed by LIR-type terminal sequences. *Bari1*-like elements with a similar structure have been identified in the genomes of *D*. *bipectinata*, *D*. *biarmipes*, *D*. *suzukii*, *D*. *takahashii* and previously in *D*. *ananassae* [9] but all them are inactive, while the *Bari1*_*D*.*rho* can be considered as a putatively active element (although the presence of the very last two nucleotides in the right TIR has not been confirmed, see also S2 Table).

The *D*. *miranda Bari* element encodes a *Bari3*-like transposase (73% similarity, see Table 1) and shows divergent terminal sequences (71.8% similarity, Table 1). A single putatively autonomous element can be found in the current genome assembly of *D*. *miranda* along with several defective elements carrying internal deletions (see S2 Table).

In *D*. *albomicans* a 109 bp long fragment can be only identified on the basis of its nucleotide similarity (75%) with the *Bari2_Dere* element (*Bari2*). This sequence, arbitrarily assumed to be a left terminus, contains the left middle (Lm) and left inner (Li) DRs, that can be easily recognized with multiple alignment analysis (Fig 2) and allow its classification in the *Bari* family, and specifically its assignment to the *Bari2* subfamily (see last paragraph of the Results section).

The putative ancestral sequence of *Bari* elements of *D*. *suzukii* and *D*. *bipectinata* element were reconstructed from truncated elements (*Bari1_Dsuz* element reconstructed from AWUT01004624 and AWUT01009036; *Bari1_Dbip* element reconstructed from contigs AFFE02004473 and AFFE02005005. See S2 Table for sequence coordinates). Both the *Bari1_Dbip* and the *Bari1_Dsuz* elements are members of the *Bari1* subfamily and possess LIR-type terminal sequences.

In *D*. *eugracilis* a low-scoring sequence contained a truncated copy of a *Bari* element with single terminal sequences with three DRs (Fig 2 and S2 Table).

While in *D*. *grimshawi* we were not able to detected elements related to the *Bari* family, in *D*. *ficusphila* and *D*. *elegans* we have identified *Bari*-related MITEs, described in a specific paragraph. In *D*. *grimshawi* the absence of *Bari*-like elements is possibly due to the actual absence of the transposon or early draft status of the genome sequencing.

### New insights into the *Bari1* heterochromatic cluster in *D*. *melanogaster*

We identified 61 independent heterochromatic *Bari1* copies in heterochromatic tandem repeat configuration. The contig JSAE01000772 contains the LTR-retrotransposon *MAX* embedded in 20 *Bari1* copies repeated in tandem. This observation matches with previous findings concerning the discontinuity in the *Bari1* repeat [17]. Two additional contigs identified in this study (ac. nos. JSAE01000400 and JSAE01000412) contain respectively 18 and 17 *Bari1* copies in tandem repeat configuration. These contigs map to the borders of the *Bari1* cluster and contain terminal copies matching also identified in previous studies [18]. We can orient of these contigs with respect to the centromere of the second chromosome considering that: 1) the *Responder* repeats, representing the major satellite DNA in this heterochromatic region [16] are abundant in the JSAE01000412 contig (388 copies); 2) *Responder* maps proximally to the centromere with respect to the *Bari* repeat as demonstrated by previous studies [15, 19]. It can be concluded that contigs JSAE01000400 and JSAE01000412 map, respectively, distally and proximally to the centromere of the second chromosome.

Scaffold JSAE01000412 contains two adjacent truncated *Bari1* copies (position 169962–173070, corresponding to elements #31 and #32, in S2 Table). We hypothesized that either inter-monomer recombination or unequal crossing over events involving adjacent or non-adjacent copies of the cluster and occurring at short homologous sequences, as shown in S2 Fig (panel A). Comparison of these two adjacent elements to two canonical adjacent *Bari1* heterochromatic copies (i.e. containing two full-length *Bari1* elements carrying the deletion of the fist two nucleotides) revealed a 340 bp deletion flanked by short stretches of identical sequences that could mediate recombination leading to the observed deletion (S2 Fig, panel B).

The last *Bari1* copy of the cluster toward the centromere of chromosome 2 represents an additional defective *Bari1* element. This truncated copy retains only 16 terminal nucleotides of the right TIR, previously described in [18]. A possible origin of the terminal copies of the h39 cluster, linked to an aberrant activity of the transposase has been previously proposed [19].

The last worth-noting scaffold (JSAE01000184) contains six *Bari1* copies arranged in a tandem repeat configuration, embedded in more than 200 kb of repetitive DNA. RepeatMasker analyses revealed the presence of transposable elements belonging to the R1 and the R2 class, typically inserted into rDNA sequences as well as few unmasked sequences related to rDNA genes (at least 4 copies). As previously suggested, this short *Bari1* cluster could either map in a distinct h39 sub-region or could be located on the X chromosome, in the region of the nucleolar organizer region. Based on the recent findings that no rDNA repeat nor type 1 rDNA insertions exist outside the nucleolar organizer regions *D*. *melanogaster* [51], it could be concluded that the small *Bari1* cluster may be located in the heterochromatin of the X or Y chromosomes, where the nucleolar organizers map.

### MITE elements related to *Bari* transposons

The tBLASTn strategy described above fails in detecting *Bari* copies carrying extensive deletions of the transposase gene, as can be observed in MITEs. Dias and Carareto recently described two MITE elements related to *Bari1* (*msechBari1* and *msechBari2*, hereafter *Bari_Dsec_MITE1* and *Bari_Dsec_MITE2*) present in multiple copies in the genome of *D*. *sechellia* [21], therefore, the existence of *Bari*-related MITEs in other species cannot be excluded. To test this possibility we performed a BLASTn-based search of *Bari*-like sequences in the species in which *Bari*-like elements were not found on the first attempt (namely *D*. *ficusphila*, *D*. *elegans* and *D*. *grimshawi*). The genomes of *D*. *ficusphila* and *D*. *elegans* harbor 36 and 4 *Bari*-related MITEs respectively. We, therefore, searched for *Bari*-related MITEs in all the available sequenced Drosophila genomes. We identified and annotated 152 copies of *Bari*-related MITEs in nine genomes, including *D*. *sechellia* (Table 1 and S3 Table). Names were assigned according to the rule given in the Materials and Methods section. In the genomes of *D*. *simulans*, we identified a *Bari*-related MITE identical in sequence to *Bari_Dsec_MITE1* of *D*. *sechellia* [21]. However, while *Bari_Dsim_MITE* is present as single copy element in the genome of *D*. *simulans*, more than twenty *Bari_Dsec_MITE1* copies are found in *D*. *sechellia* ([21] and this study, Table 1). In *D*. *melanogaster*, we identified two *Bari*-related MITE families based on their length. The *Bari_Dmel_MITE1* family is an 116 bp long element with nearly identical, 46 bp long, terminal inverted repeats. The *Bari_Dmel_MITE2* family contains longer elements (692 bp) and has 50 bp long terminal inverted repeats. *D*. *suzukii* harbors a single family of *Bari*-related MITEs, nearly 500 bp long, sharing high sequence similarity if compared to each other. A retrospective analysis of the single copy element previously identified in *D*. *yakuba* [9] led us to conclude that it can be classified as a *Bari*-related MITE. A peculiar feature of this MITE consists in the presence of two DR sequences in the left terminus while all the other *Bari*-related MITEs display a single DR in each terminus.

**Fig 4:**
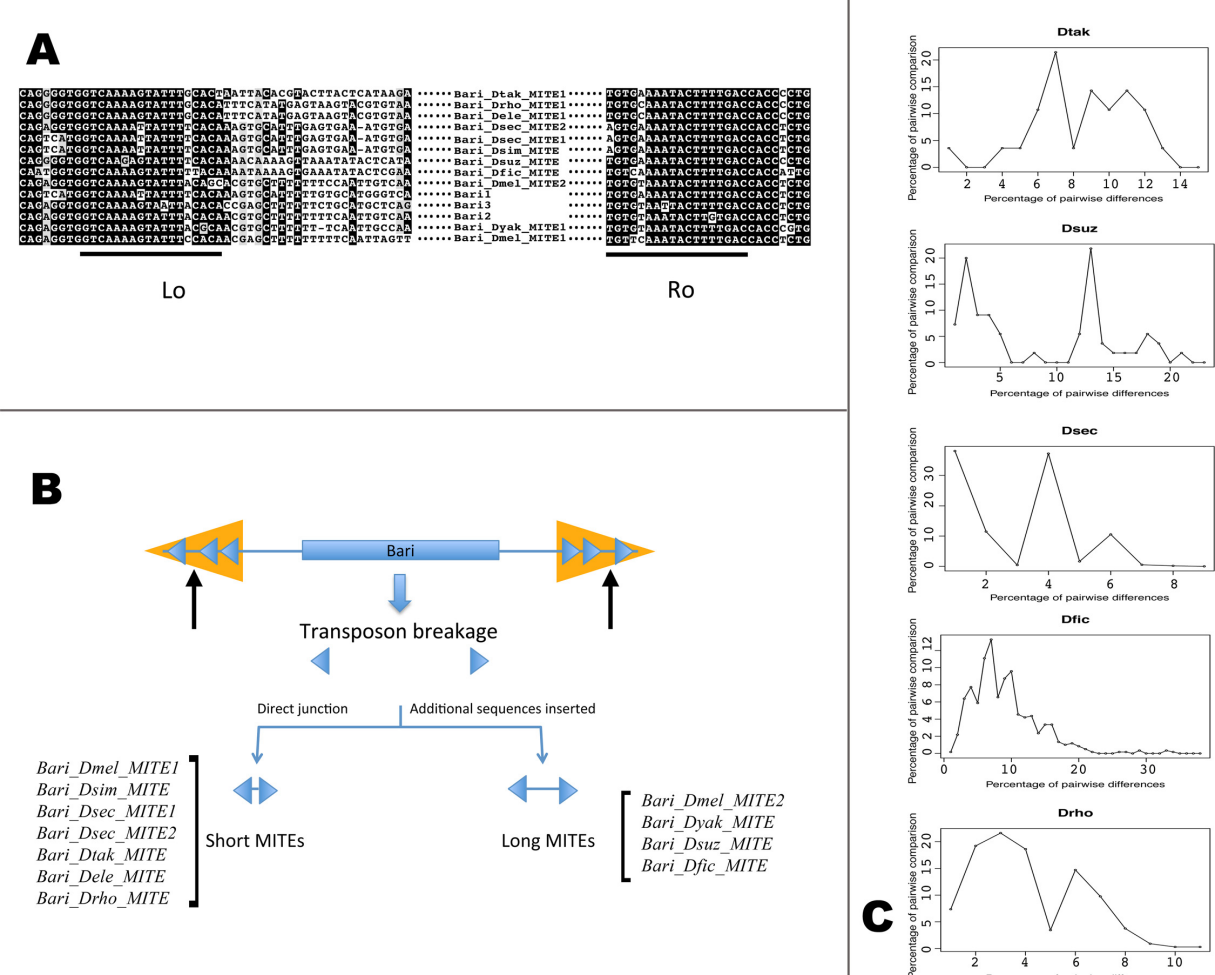
The *Bari*-derived MITEs. (A) Multiple alignment slices relative to the left and right terminal sequences of the *Bari*-related MITEs compared to the canonical elements *Bari1*, *Bari2* and *Bari3*. Lo and Ro are Left outer and Right outer Direct Repeats respectively. (B) Possible origin of *Bari* related MITEs through internal deletion of a functional element with breakpoints between the Lo-Lm and Rm-Ro DRs (see arrows). (C) Pairwise nucleotide diversity distribution in *Bari*-related MITE elements. Species displaying a unimodal distribution of the sequence similarity (Drho, Dfic and Dsec) and species displaying bi- or multi-modal distribution (Dsuz and Dtak) of the sequence similarity are shown. Hartigan dip test for unimodality / multimodality: Drho p = 0,598; Dfic, p = 0,2135; Dsec, p = 0,4119; Dsuz, p = 0,01805; Dtak, p = 0, 01374. H0 is unimodality.

An overall comparison of the terminal sequences of the *Bari*-related MITEs with the terminal sequences of *Bari1_Dmel*, *Bari2_Dere* and *Bari3_Dmoj*, representative of the *Bari1*, *Bari2* and *Bari3* subfamilies respectively, is shown in Fig 4A. All the terminal sequences of *Bari*-related MITEs are very similar to each other and share similarity with the corresponding full-length elements. *Bari*-MITE sequences contain an intervening sequence between the two terminal that can be either short or long but with poor sequence similarity if compared to reference elements (S3 Fig), suggesting that complex mechanisms might cause their origin from master elements (Fig 4B). Although the sequence similarity at the terminal sequences level suggests that these MITEs belong to the *Bari* family, no obvious ancestor can be inferred using this approach. MITEs with identical sequence have been identified in different species (i.e. *Bari_Dsec_MITE1* and *Bari_Dsim_MITE*) while in other cases MITEs from different species have a very similar sequence (*Bari_Drho_MITE* and *Bari_Dtak_MITE* share 75 out of 78 nucleotides).

To investigate the mechanisms of MITEs expansion, we postulated that the analyzed MITEs derived from the same ancestor and performed pairwise nucleotide diversities analysis of these elements (Fig 4C). Rogers and Harpending [52] reported that episodes of population growth leave a characteristic signature in the distribution of nucleotide differences between pairs of individuals, and this concept was used to describe the mode of amplification of MITEs in *Oryza sativa* [53] [54]. We adapted this type of analysis to the *Bari*-derived MITEs and statistically evaluated whether the wave-like form of the histograms could fit unimodal distribution. Our data do not allow rejection of unimodality in three species (*D*. *sechellia*, *D*. *ficusphila*, *D*. *rhopaloa*; Hartigan dip test for unimodality: Drho p = 0,598; Dfic, p = 0,2135; Dsec, p = 0,4119) suggesting single burst of transposition in these genomes. In the genomes of *D*. *suzukii* and *D*. *takahashii*, a multimodal distribution of the pairwise differences (Hartigan dip test for unimodality: Dsuz, p = 0,01805; Dtak, p = 0, 01374) suggests that multiple rounds of MITEs transposition occurred.

### Bari-like elements and their target site selection preferences

**Fig 5:**
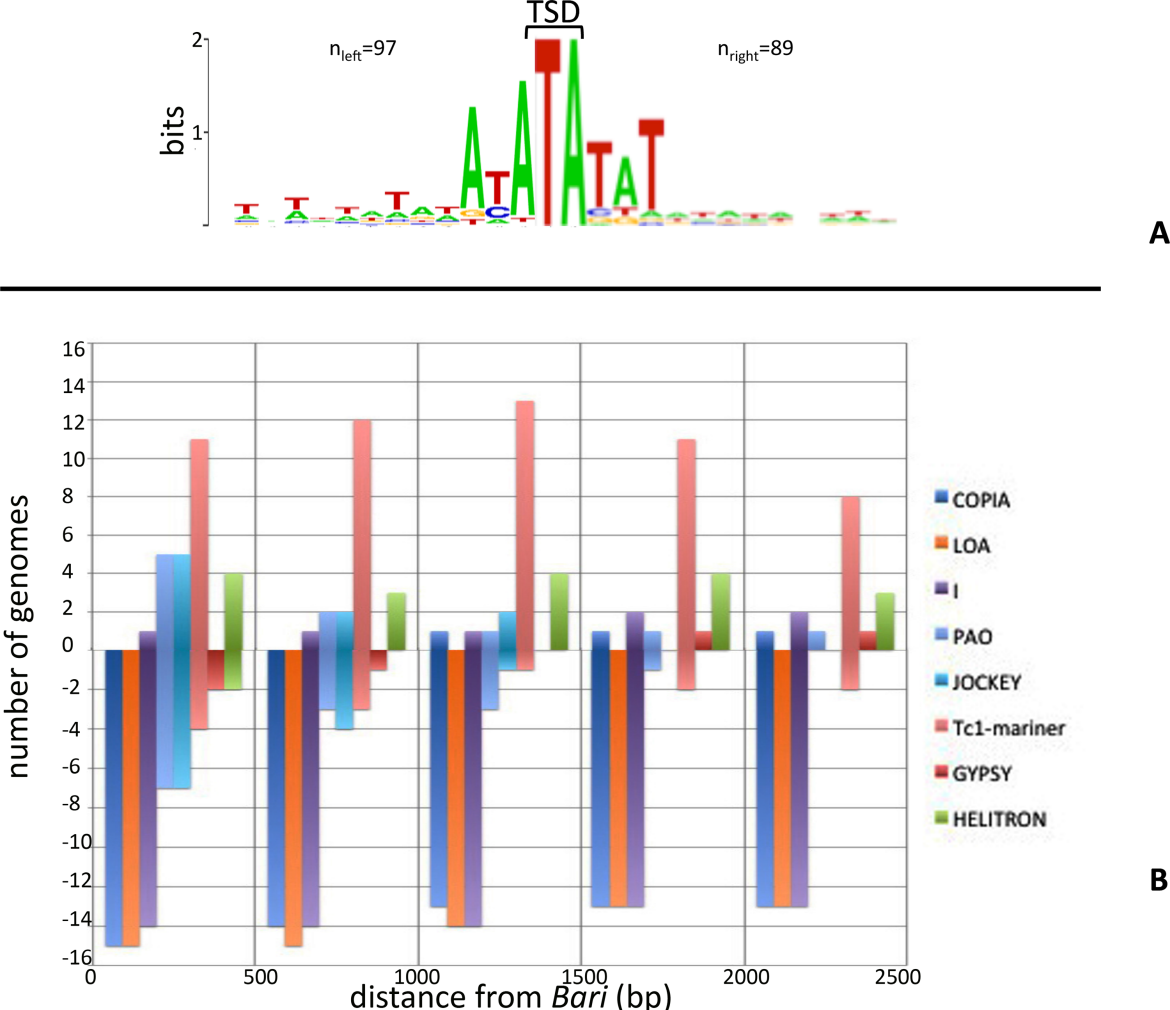
Target site preferences of *Bari* elements. (A) WebLogos showing the preferred target sequence and the target site duplicated upon integration (TSD) of *Bari* elements. Results obtained from single species are shown in S4 Fig. The number of flanking sequences analyzed (n) is given. (B) Correlation between *Bari* elements and members of different transposon super-families. Y-axis indicates the number of genomes in which a given transposon family is significantly found associated (positive values) or is significantly under-represented (negative values) at a given distance (X axis) from the *Bari* elements. The position of the *Bari* element is conventionally fixed at the origin of X-axis. Results obtained from single genome analyses are shown in S5 Fig.

To gain insight into the choice of integration target sites for *Bari*-like elements, we performed flanking sequences analyses. It is well established that *Tc1-mariner* elements integrate themselves into the TA target, which is duplicated upon element insertion [10]. Multiple alignment of 15 bp long sequences encompassing the *Bari* elements insertion sites we identified, showed that this is also true for *Bari* elements, as also highlighted by the WebLogo analyses (Fig 5A).

Furthermore, the *Bari* transposase targets AT-rich sequences irrespective of the *Bari* subfamily and of the host species, as can be observed in S4 Fig.

We further investigated the target site preferences of *Bari*-like elements on a larger sequence scale by analyzing the sequence context in which *Bari* transposons insert themselves.

The presence of transposable elements belonging to three LTR-retrotransposons superfamilies (*Copia*, *Gypsy* and *PAO*), three non-LTR retrotransposons superfamilies (*LOA*, *I* and *Jockey*), the *Tc1-mariner* superfamily and to the *Helitron* superfamily, was annotated within a 2,5 kb long sequence flanking upstream and/or downstream the *Bari* elements in each species analyzed. To assess whether the observed distribution of transposable elements within the neighborhoods of *Bari*, significantly differ from their genomic distribution in the respective genomes, we collected 1000, non-overlapping, random sequences, of the same length (2,5 kb), irrespective of their gene content (S1 File), and recorded the occurrence of tested TEs. This analysis was performed in genomes containing a minimum of 4 *Bari* insertions (an arbitrarily chosen threshold), resulting in 15 investigated genomes (Fig 5B and S5 Fig). In these genomes, the *Bari* transposon insertions occur in genomic loci depleted in *Copia*, *LOA* and *I* elements and enriched in *Tc1-mariner* elements. In 5 genomes we also found a significant tight association (within 500 bp from the *Bari* elements) with elements of the *Jockey* and *PAO* families; however in 7 genomes, taking into account the same sequence range, *Bari* elements insertions occur in genomic regions significantly depleted in *Jockey* and *PAO* elements. *Gypsy*-like retrotransposons do not display significant enrichment or depletion within the 2,5 kb range considered. *Helitron*-like elements also significantly co-occur within 2,5 kb from *Bari* elements.

### Evolutionary analyses of *Bari* transposons

**Fig 6:**
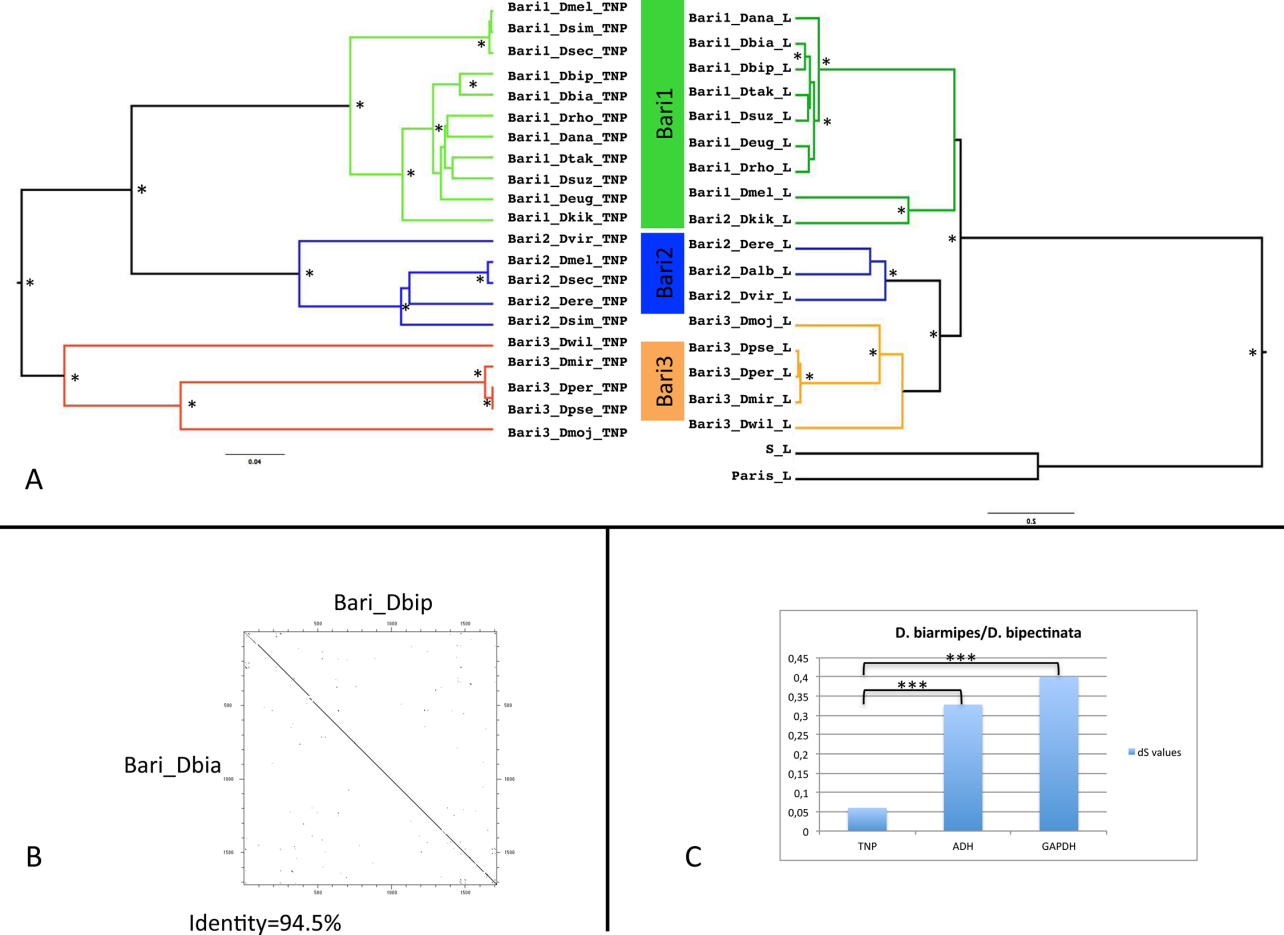
Evolution of the *Bari* elements. (A) Bayesian phylogenetic trees of *Bari*-like elements. The tree on the left was built using the transposase genes alignment and the GTR**+**G model while the tree on the right was built using the left terminal sequences containing the DRs of elements and the HKY+G model. Asterisks next to the branches denote posterior probability values greater than 0,9. The color code legend used to indicate the *Bari* three subfamilies is shown between the two trees. B) Dot plot matrix (window = 13; stringency = 11) showing the similarity of two *Bari* elements in *D*. *biarmipes* and *D*. *bipectinata*. The percent identity between the two sequences is also shown. C) Comparison of dS values of the *Bari* transposase-coding region with the dS values of two nuclear genes in *D*. *biarmipes* and *D*. *bipectinata*. (***; p<0,005).

We multi-aligned the transposase-coding sequences from 16 representative *Bari* copies and built a phylogenetic tree using Bayesian inference methods. The phylogenetic tree created recovered three clades with significant statistical support (Fig 6A, left tree) and corresponding to the previously described *Bari1*, *Bari2* and *Bari3* clades [9]. This analysis placed the newly identified *Bari* elements of *D*. *suzukii*, *D*. *takahashii*, *D*. *rhopaloa*, *D*. *bipectinata*, *D*. *biarmipes*, *D*. *kikkawai* and of *D*. *eugracilis* in the *Bari1* clade while the element of *D*. *miranda* cluster together with other *Bari3* elements.

We also performed phylogenetic analyses based on the terminal sequences of *Bari*-like elements. Roughly 250 nucleotides containing the three DRs from the left terminal sequence of *Bari*-like elements were analyzed in a Bayesian tree (Fig 6A, right tree). The phylogenetic tree obtained displays a topology similar to that obtained for the transposase gene-based tree, with three distinct and well-supported clades consisting of the three *Bari* subfamilies.

A careful inspection of the transposase-based phylogenetic tree revealed an inconsistency in a well-supported *Bari1* sub-clade if compared to the phylogeny of species (Fig 1A). The *Bari1_Dbia* and *Bari1_Dbip* elements are indeed very close to each other in the transposase tree, while their respective host species, *D*. *biarmipes* and *D*. *bipectinata*, are more distantly related, being members of the suzukii and ananassae subgroups respectively (Fig 1A). These elements share high sequence similarity (Fig 6B) and are part of a highly supported clade in the tree. In order to discern if the incongruences observed in the phylogeny of *Bari*-like elements and host species were due to vertical transmission or horizontal transfer, the divergence at synonymous sites (dS), taken as a measure of neutral evolution in the absence of a strong codon usage bias, was compared between TEs and *Adh* or *Gapdh1* genes of the two species. Fisher exact test-based comparisons were performed to verify whether the dS of the *Bari*-like elements was significantly lower than the dS of nuclear genes [55]. The dS of *Bari* elements (0,0603) was significantly different (p<0,005) if compared to dS of *Adh* (0,3281) or *Gapdh1* (0,4006) genes in these species (Fig 6C), supporting the hypothesis of horizontal transfer of *Bari*-like elements between these species.

## Discussion

This work aimed to identify *Bari* transposon insertions in the sequenced Drosophila genomes, a step forward in the annotation of the complete set of TEs insertions in these genomes. We have annotated 401 copies (S2 and S3 Tables) of *Bari*-like transposon in the genomes of 22 Drosophila species out of 23 available sequenced genomes. Although this data may seem as an overestimation of the actual number of *Bari* elements in the analyzed species, we expect that elements being missed due to the early draft status of some genome assembly (especially in the heterochromatin) would somewhat compensate any number discrepancies. Importantly, Southern blot experiments testing the genomic distribution of *Bari* elements in some of the genomes analyzed in this work [56, 57] are in substantial agreement with our sequence analyses, suggesting that *Bari* is present in low copy number in these genomes and that *D*. *melanogaster* is the only species (among those studied) containing heterochromatic clusters.

This work provided a number of novel insights into the biology of the *Bari* transposon’s family:

1. high sequence variability of the terminal sequences coupled with high conservation of the DRs and wide diffusion in the Drosophila species’ genomes.
2. MITE-like forms of this transposon, arising occasionally during the species evolution.
3. strong target site preference for AT-rich DNA regions, which is also the case for other Tc1-mariner elements, that are instead avoided by class I transposable elements.
4. occasional horizontal transfer events involving *Bari*-like elements.

### Bari structural diversity

Transposons structural diversity is an obvious evolutionary phenomenon if large families or super-families of mobile elements are taken into account [58]. However, the emergence of structural variants can also be detected in small transposons’ families, such as the *Bari* family.

The dynamic processes leading to TEs replication, amplification, degradation and elimination from a given genome are difficult to be elucidated. However the graphic representation of the deterioration pattern of TEs proposed by Fernandez Medina et al., [58] can be used to shed light into this task. Our results (Fig 3) suggest that *Bari* elements have undergone deterioration in different ways depending on the host genome. Point mutations and indels probably weighted differently in producing non-functional *Bari* copies during evolution, because mutations in the terminal sequences are more tolerated if compared to the coding sequence. Despite some degree of diversification observed at the terminal sequence level (Fig 3 and S1 Fig), a strong conservation of the DR sequences in all elements’ subfamilies from all species, including those containing only MITEs (Fig 2A and Fig 4A) suggests that these sequences may have acquired a potential role during the evolution. The observed differences in the p-values of the terminal sequences can be ascribed either to the transposition activity of *Bari* elements or to the presence of functional elements in the terminal sequences. For example *D*. *mojavensis* contains 8 potentially active and 7 inactive *Bari3*-like elements and a median p-distance value of 0 for both terminal sequences, while *D*. *miranda* contains 1 potentially active and 4 inactive *Bari3* elements and a median p-distance values of 0,171 and 0,06 for left and right terminal sequences respectively. Similarly *D*. *melanogaster* contains 4 potentially active and 1 inactive *Bari1*-type elements with a median p-distance value of 0 for both terminal sequences while *D*. *simulans* contains two potentially active elements with a median p-distance value of 0,01 for both terminal sequences. In conclusion, it can be speculated that species displaying lower p-distance values host active *Bari* elements, which produce identical copies upon transposition thus lowering the p-distance. By contrast, species with higher p-distance values contain many dead or almost dead elements, which rapidly accumulate more mutations and become divergent in sequence. However, we have evidence of *Bari* transposition only in *D*. *melanogaster* [13] and in *D*. *mojavensis* [14], needing additional studies in order to establish if *Bari* is active in non-model species. The presence of functional elements could also explain the differences observed in p-distance values between the terminal sequences if compared to the CDS. While both the left and right termini contain DRs able to bind the transposase, the left terminus might carry the promoter or part of it, and the right terminus might contain important signals for the termination of transcription. Interestingly in the context of the terminal sequences the DRs appear strongly conserved in sequence (Fig 2) even in species lacking active *Bari* elements. Based on this it could be speculated that DRs have acquired a new function (e.g. production of siRNA, binding of different protein partner etc) in the genomes where *Bari* elements have been completely inactivated.

There is evidence from previous studies [9] suggesting a well-defined structural vs phylogenetic relationship among *Bari*-like elements. While elements of the inactive *Bari2* subfamily contain LIR-type terminal sequences, elements of the *Bari1* and *Bari3* subfamilies usually harbor SIR and LIR respectively. The non-autonomous *Bari1* elements identified in *D*. *ananassae* represent an exception as they harbor LIR [9]. In this paper, we show the existence in *D*. *rhopaloa* of potentially active *Bari1* elements containing identical LIR, which is a previously unreported feature in the *Bari1* subfamily that increases the diversity among *Bari* elements. In addition we identified *Bari1*-type elements with LIRs in *D*. *bipectinata*, *D*. *biarmipes*, *D*. *suzukii* and *D*. *takahashii*, but in these species all the *Bari* elements are inactive. To date, the *Bari1_Drho* represents the only potentially active *Bari*-1 type element with LIRs. It was previously suggested that the identical LIRs might represent the first stage of the evolution of the terminal repeats of *Bari-*like elements [9]. The LIR structure may subsequently evolve into SIR structures as a consequence of the intrinsic instability associated with the long terminal repeats structure [9]. This hypothesis was best fitting with the *Bari3* subfamily, in which the *Bari3_Dmoj* represents a “young” *Bari* element having perfect LIR and the *Bari3_Dper* and *Bari3_Dpse* elements, representing older elements that are going to lose the similarity between their TIRs. The same hypothesis can be now formulated for the *Bari1* subfamily, where SIR- and LIR-containing elements were previously identified. The two TIRs of the *Bari_Dana* element indeed share 94% similarity and the two TIRs of the *Bari1_Drho* element reported here are identical to each other. In this view the *Bari1_Drho* element represents a young *Bari1*-type element, still awaiting the divergence of its terminal sequences. This conclusion raises further questions concerning the mechanisms that generate *Bari1*- and *Bari3*-type elements with perfectly matching terminal sequences, which are still to be identified.

The powerful sequencing technologies developed in the last years facilitate the molecular determination of repeat-rich genomic such as the h39 heterochromatic region of *D*. *melanogaster* which hosts roughly 80 clustered copies of *Bari1* and the *Responder* satellite. Our detailed analysis allowed orienting the *Bari1* cluster with respect to the centromere of the second chromosome, thanks to the presence of the *Responder* repeats in one of the sequences flanking the *Bari* cluster.

It was previously observed that the *Rsp* satellite displayed an extreme quantitative and structural variability while *Bari1* cluster showed remarkable homogeneity [15]. The occurrence of recombination events between clustered copies of *Bari1*, as inferred from our data, suggests that this transposon cluster could be subjected to expansions or contractions as observed for other complex DNA repeats over evolutionary time. Our sequence analyses of heterochromatic copies of *Bari1* in *D*. *melanogaster* support the hypothesis that an additional *Bari1* cluster might exist outside the h39 region of the mitotic chromosomes, specifically on the X or Y chromosome, since it is associated with DNA sequences specific of the Nucleolar Organizer Region (rDNA and *R1* element insertions [51]).

The presence of such kind of clusters in a single Drosophila species (i.e. *D*. *melanogaster*) is peculiar and it could be speculated that the reiterated formation of clusters, might depend on species-specific host factors contributing to an error-prone activity of the transposase. In addition resolving the structure of heterochromatic genomic blocks, rich in transposable elements would help understanding important regulatory loci, as reported for the *flamenco* locus in *D*. *melanogaster* [59].

### MITEs related to *Bari* elements

Miniature Inverted-repeat Transposable Elements (MITEs) are non-autonomous, short repeats that mobilize within the host genome even without the potential to encode the key proteins responsible for their mobilization (i.e. the transposase). MITEs are generally smaller than 600 bp with few exceptions, have conserved TIRs, a target site preference, do not display coding potential, are AT-rich and amplified within the host genome [60]. In general, they are supposed to originate by deletions internal to autonomous elements, which leave untouched the TIRs and, sometimes, just portions of the transposase. This origin supports the hypothesis of their mobilization *in trans* by a transposase encoded by a full-length element [61]. In addition to the previously described *Bari*-related MITEs in the genome of *D*. *sechellia* [21], we identified this element type in eight additional Drosophila species. These novel *Bari*-related MITE sequences match the definition of MITEs; however some of them (*Bari_Dmel_MITE2*, *Bari_Dsim_MITE*, *Bari_Dyak_MITE)* lack genomic amplification while others show only modest amplification (*Bari_Dmel_MITE1*, *Bari_Dtak_MITE*, *Bari_Dele_MITE)*. By contrast *Bari_Dsec_MITE1*, *Bari_Dsec_MITE2* [21], *Bari_Dfic_MITE* and *Bari_Drho_MITE* are quite abundant in the respective genomes. It is possible that MITEs in *D*. *melanogaster*, *D*. *simulans* and *D*. *takahashi* could be in a very initial stage of their amplification. Alternatively, they might represent the product of abortive amplification.

Wallau et al., [62] recently described 27 independent sub-lineages of *mariner*-derived MITEs in 20 Drosophila species, which have internal sequences and TIRs similar to the sequences of the full-length copies and a typical size of 900–1000 bp with few exceptions. *Bari*-related MITEs are shorter than the *mariner*-related and possess internal regions not easily comparable with autonomous elements, reflecting a more complex rearrangement differing from abortive gap repair [63]. Furthermore, *Bari*-derived MITEs can be short (less than 120 bp) or long (greater than 120 bp and less than 700 bp) in sequence, and the intervening sequence between the TIRs, where present, is apparently unrelated to *Bari* elements (S3 Fig). Contrarily to the *mariner* MITEs [62], *Bari*-related MITEs apparently originated by deletions in the same point, with the possible exception of the *Bari_Dyak_MITE*. It can be hypothesized that *Bari*-related MITEs originated through internal deletion of a master element, with the deletion breakpoints occurring between the Lo-Lm and between Rm-Ro direct repeats (Fig 4B), which has been possibly followed either by direct junction of the broken extremities or by the addition of unrelated sequences (Fig 4B). Finally, as possible explanations for the presence of MITEs in those species lacking full-length elements (namely *D*. *yakuba*, *D*. *ficusphila* and *D*. *elegans*), it is plausible that these MITEs derived from the ancestral transposition of full-length elements, followed by generation of MITEs and elimination of the original founders. Thus, the single elements identified are just the relic of this elimination. Expansion of MITEs has probably occurred one or multiple times within the host genomes as suggested by sequence similarity distribution (Fig 4C). Notably, the *D*. *sechellia*, *D*. *suzukii D*. *ficusphila* and *D*. *takahashii* genomes lack autonomous *Bari* elements, so it would be of particular interest to know if transposition events involving MITEs occurred after the elimination of autonomous elements, resulting in transposition mediated by unrelated transposases.

### *Bari* target site preferences and the physical relationships with other mobile elements

It is widely accepted that mobile elements do not integrate themselves randomly [64]. Primary and secondary DNA structure, as well as chromatin status, could affect the target site selection. Our results point out a strong preference of *Bari*-like elements for AT-rich sequences. Similar results were obtained for other *Tc1*-like transposons, like *SB* which has a preference for a palindromic AT-repeat (ATATATAT) in which the central AT is the cleaved and duplicated target [65].

In addition to the above-described analysis we have performed a larger-scale analysis aiming to the identification of TEs that preferentially insert, or preferentially avoid, the DNA neighborhood of *Bari* elements. We found that loci in which *Bari* transposons are inserted are also populated by other *Tc1-mariner* elements (p<0,05). The significant association between *Bari* elements and other transposons of the same class lead us to hypothesize a possible correlation between the DNA (or chromatin) structure and the insertion preferences of transposons belonging to the same superfamily. On the other hand, the members of the *copia*, *Jockey* and *I* families are significantly under-represented in the range of 2,5 kb around *Bari* elements in the analyzed species. *Helitron* elements also display a preference for sites near *Bari* insertions, while *gypsy*–like elements do not show significant preference/avoidance respect to *Bari* elements insertion sites suggesting that *gypsy*-like elements have a random genomic distribution. Besides its importance in identifying the structural organization of the single loci in which *Bari* elements lie, this kind of analyses would hopefully help understanding how a DNA domain is built during evolution. This is especially interesting for heterochromatic DNA blocks, which are largely composed of transposons’ arrays and can develop to master loci producing small regulatory RNAs [66, 67].

### *Bari* phylogeny inconsistencies and possible horizontal transfer events

The evolution of *Bari* elements has been extensively studied in previous works [9, 19, 57]. The non-uniform distribution of the three subfamilies across the species of the Drosophila and Sophophora subgenera was observed by Moschetti et al., [9] and explained with a distinct evolutionary history in different genomes. The overlap observed here, between the *Bari1*-*Bari2* subfamilies in some species (*D*. *melanogaster*, *D*. *simulans*, *D*. *sechellia*), might be explained hypothesizing two independent waves of genomic invasion: the first, most ancient, by *Bari2* elements which probably occurred in the ancestor of the melanogaster subgroup (comprising the melanogaster, simulans, sechellia and erecta species analyzed in this work), the second, more recent, by *Bari1* which have invaded the ancestor of the melanogaster complex (including the melanogaster simulans and sechellia species). Assuming only vertical transmission, *Bari2* elements have been inactivated while *Bari1* elements are still active in *D*. *melanogaster* and, potentially, in *D*. *simulans*. After these invasions, *Bari2* elements were probably inactivated resulting in the absence of active copies in these genomes. It would be interesting to know if *Bari2* elements are also present in non-sequenced genomes of the Drosophila subgenus, or if *Bari2* subfamily is restricted to species of the Sophophora subgenus.

The emergence of transposable elements in a genome can occur in three ways: *de novo* emergence, horizontal transfer and introgression [68]. Patchy TEs distribution, incongruent TE vs host species phylogeny and the presence of highly similar sequences in distantly related species [69] are used as proofs in support of TEs horizontal transfer. Several horizontal transfer events involving *Tc1-mariner* elements have been described so far [70]. In a recent paper, Dupeyron and collaborators described horizontal transfer events between terrestrial isopod crustaceans and hexapods involving *Tc1-mariner* elements [7]. Horizontal transfer events were also suggested for *Bari*-like elements between the sibling species *D*. *melanogaster* and *D*. *simulans* as reported by former studies [71] [69]. Here we present evidence of horizontal transfer events involving *Bari*-like elements between *D*. *bipectinata* and *D*. *biarmipes*. Although these two species diverged at least 27 million years ago [25], *Bari* elements within the respective genomes are nearly identical. Analysis of synonymous substitutions differences between transposases and host genes suggest that horizontal transfer occurred, which explain the incongruences observed in the phylogenetic trees and the high similarity between the *Bari* elements in these species (Fig 6A and 6B). The geographic distribution of the Drosophila species involved in transposon horizontal transfer events supports this possibility, as these two species coexist in the Indian subcontinent. Very recently Wallau et al., reported the same horizontal transfer event described here by applying a sophisticated and statistically supported method, called VHICA, [72] which used 50 orthologous, vertically transmitted genes, as a reference set to infer horizontal transposon transfer of *Bari* between *D*. *biarmipes* and *D*. *bipectinata*. Other horizontal transposon transfer events involving different species have been also detected using this method [72] suggesting that complex evolutionary mechanisms have originated the actual distribution of *Bari* elements, complicating the inference of their evolutionary history.

## Conclusions

Our annotation and analyses of 401 insertions unveiled sequence and structure variability of *Bari*-like elements in the sequenced genomes of Drosophila species. Besides the dynamic structure of the TIRs and the presence of active and inactive elements in the three *Bari* subfamilies, we detected the presence of MITEs derived from *Bari* elements in 9 Drosophila species, suggesting that the generation of such inactive form can be considered as a common event in this family. The analysis of genomic sites targeted by *Bari* transposition showed that the same sites are also preferred or avoided by other mobile elements, and this may be important to understand how transposons model genomic domains. Finally our phylogenetic analysis showed that three subfamilies (*Bari1*, *Bari2* and *Bari3*) can be recognized both in TIR- and transposase-based phylogenetic trees and that a previously unreported horizontal transfer event has probably occurred between *D*. *biarmipes* and *D*. *bipectinata*.

## Supporting Information

S1 Fig P-distance analyses and deterioration profiles of Bari elements in all the analyzed species. (TIF)

S2 Fig Detection of a recombination site generating a deletion in the heterochromatic cluster. **A.** Possible molecular mechanism generating the observed adjacent heterochromatic copies carrying deletions of terminal sequences. (**B**) Global alignment (Needleman-Wunsch) of two adjacent canonical *Bari1* heterochromatic copies and the detected copies carrying a deletion (elements #31 and 31, S2 Table). The sequences of the two adjacent monomers are shown in red and blue color fonts. Deletion breakpoints, corresponding also to homologous sequences involved in inter-monomer recombination events, are highlighted in yellow.
(PDF)

S3 Fig Multiple alignment of representative MITEs and reference *Bari* elements. (PDF)

S4 Fig WebLogo analysis. Fifty bp upstream or downstream the *Bari* elements in 8 Drosophila species were analyzed. The number of sequences analyzed is reported (n).
(TIF)

S5 Fig Transposons co-occurrence near the insertion site of *Bari* elements in single species. The red-boxed area in each plot indicates area of significant (p<0,05) enrichment (positive values) or depletion (negative values) of the analyzed transposable elements’ super-families in the proximity of *Bari* elements in a sequence range of 2,5 kb. X axes report p value. Y axes report the distance from origin in bp.
(PDF)

S1 File Accession numbers and coordinates of 1000 randomly selected sequence used as control dataset in flanking sequences analysis. (XLSX)

S1 Table Genome assemblies used for analyses. (PDF)

S2 Table Complete dataset and features of *Bari* elements annotated in each species analyzed. (XLSX)

S3 Table Complete dataset and features of *Bari*-related MITEs annotated in each species analyzed. (XLSX)

S4 Table Tukey test and Kruskal-Wallis test results. (PDF)
