# Supplementary figures and images for: Identification of Bari Transposons in 23 Sequenced Drosophila Genomes Reveals Novel Structural Variants, MITEs and Horizontal Transfer
Source: PLoS One. 2016 May 23;11(5):e0156014. doi: 10.1371/journal.pone.0156014 (PMC4877112; doi:10.1371/journal.pone.0156014)

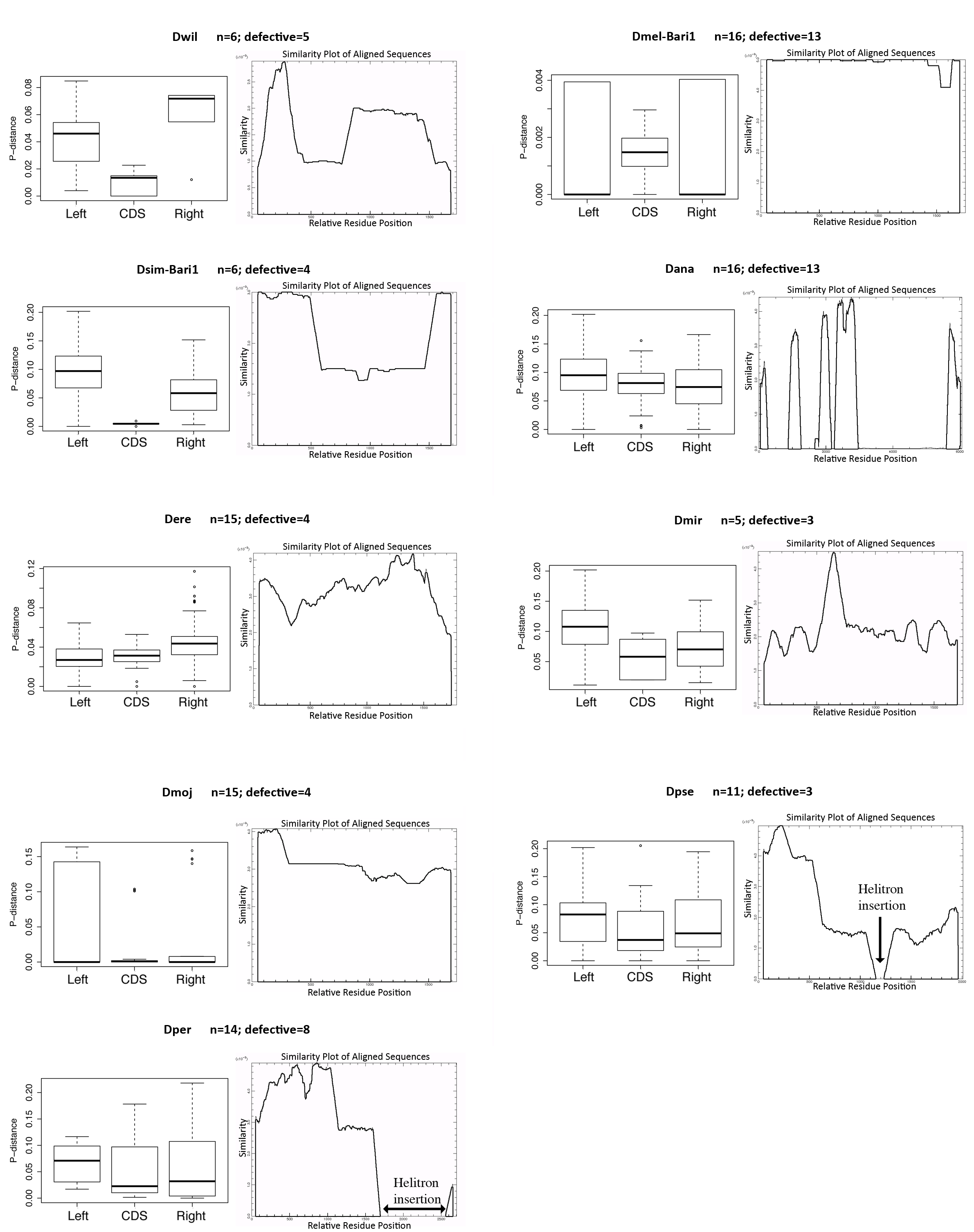

Supplement: S1 Fig — (TIF) [file pone.0156014.s001.tif]

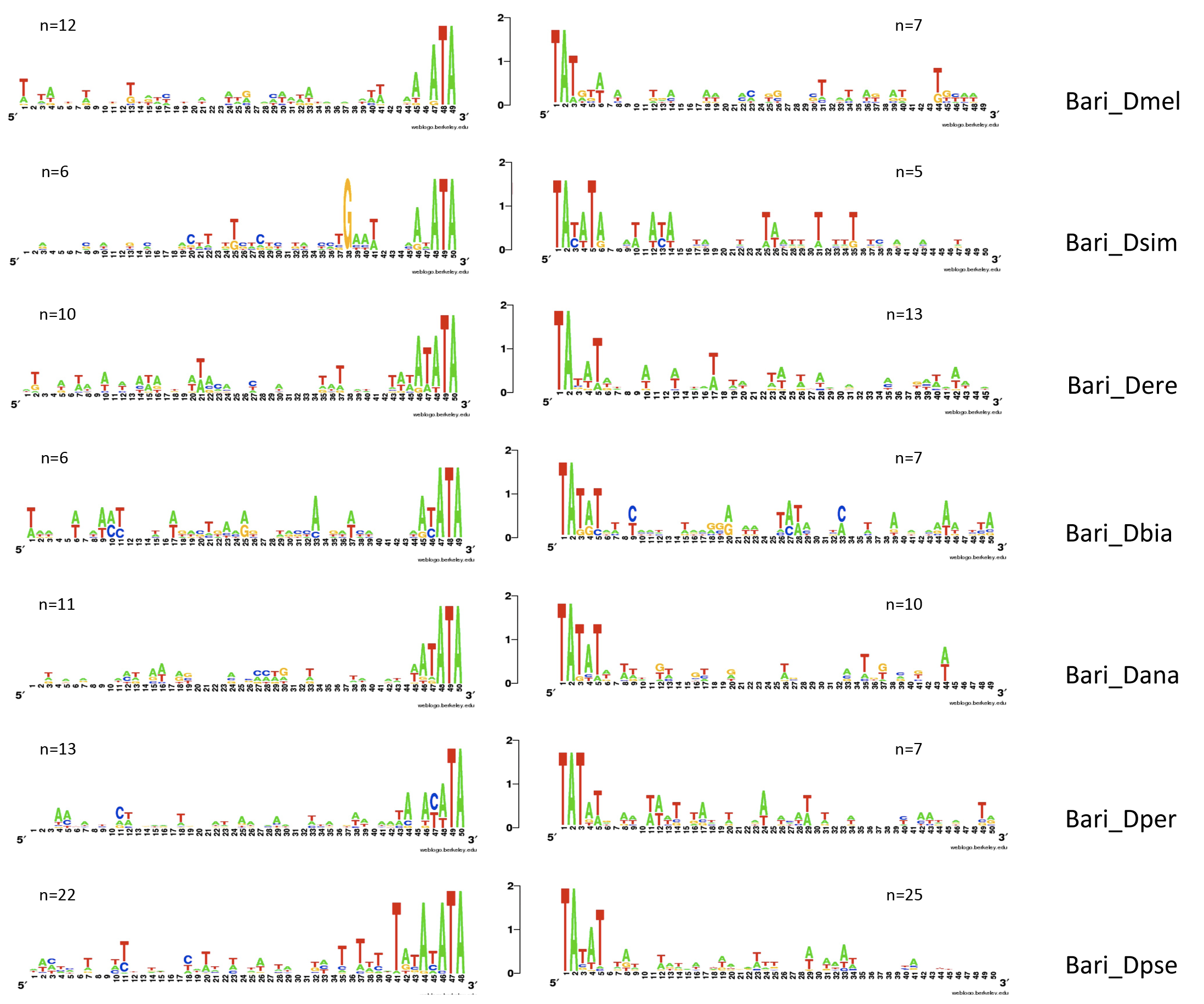

Supplement: S4 Fig — Fifty bp upstream or downstream the Bari elements in 8 Drosophila species were analyzed. The number of sequences analyzed is reported (n). (TIF) [file pone.0156014.s004.tif]
