# Supplementary material for: Identification of Bari Transposons in 23 Sequenced Drosophila Genomes Reveals Novel Structural Variants, MITEs and Horizontal Transfer
Source: PLoS One. 2016 May 23;11(5):e0156014. doi: 10.1371/journal.pone.0156014 (PMC4877112; doi:10.1371/journal.pone.0156014)
Supplement: S2 Fig — A. Possible molecular mechanism generating the observed adjacent heterochromatic copies carrying deletions of terminal sequences. (B) Global alignment (Needleman-Wunsch) of two adjacent canonical Bari1 heterochromatic copies and the detected copies carrying a deletion (elements #31 and 31, S2 Table). The sequences of the two adjacent monomers are shown in red and blue color fonts. Deletion breakpoints, corresponding also to homologous sequences involved in inter-monomer recombination events, are highlighted in yellow. (PDF) [file pone.0156014.s002.pdf]

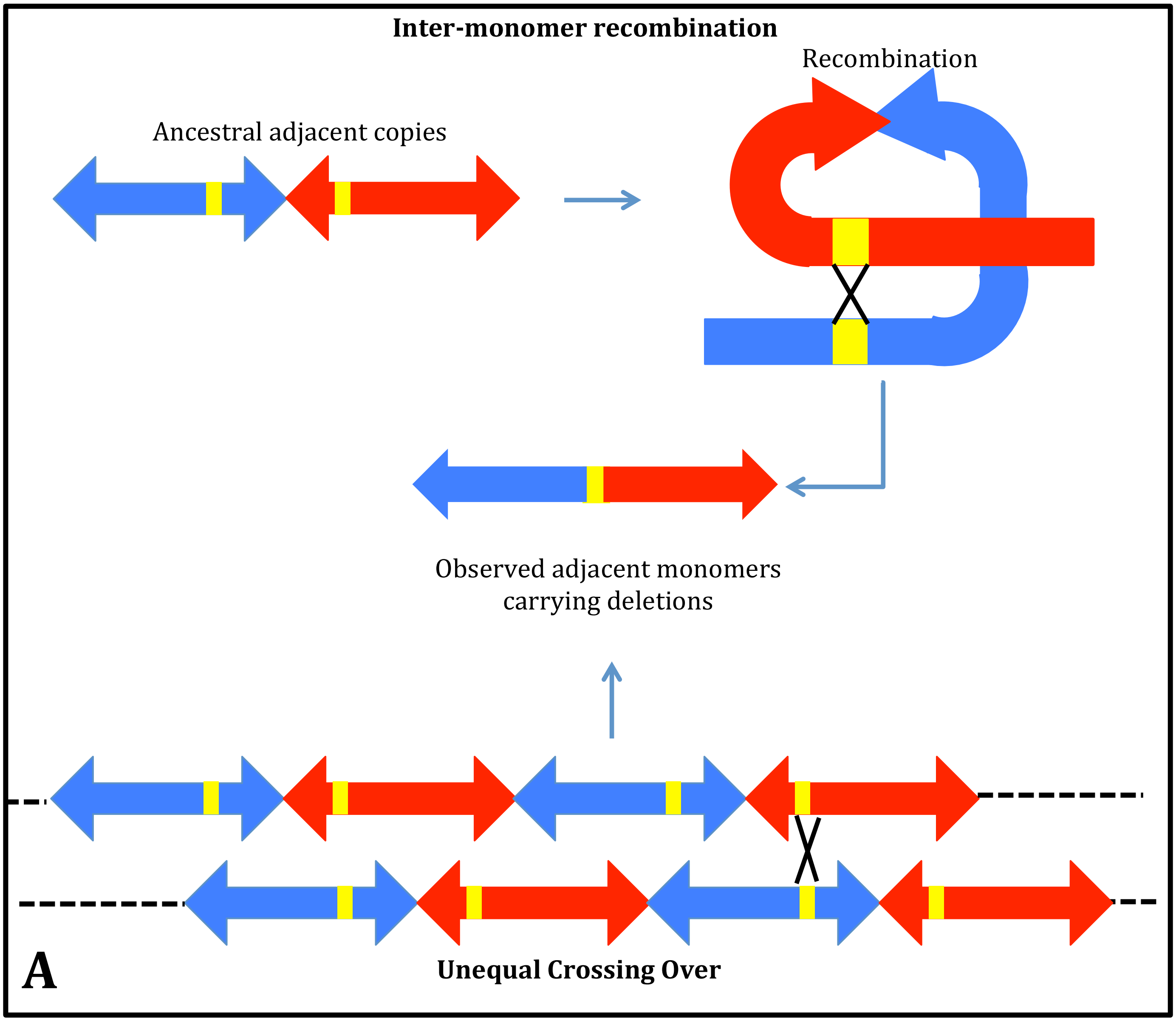

Inter-monomer recombination
Recombination
Ancestral adjacent copies
Observed adjacent monomers carrying deletions
A
Unequal Crossing Over

B

```
                10        20        30        40        50        60
Bari1   GTCATGGTCAAAATTATTTTCACAAAGTGCATTTTTGTGCATGGGTCACAAACAGTTGCT
        ::::::::::::::::::::::::::::::::::::::::::::::::::::::::::::
dimer   GTCATGGTCAAAATTATTTTCACAAAGTGCATTTTTGTGCATGGGTCACAAACAGTTGCT
                10        20        30        40        50        60

                70        80        90        100       110
Bari1   TGTGCAGCAAGTGGGGGGAGGTGAAATGCAAAAAA-CTTTTGCTTTTGCAAATTCAAAC
        :::::::::::::::::::::::::::::::::::: :::::::::::::::::::::::
dimer   TGTGCAGCAAGTGGGGGGAGGTGAAATGCAAAAAAAACTTTTGCTTTTGCAAATTCAAAC
                70        80        90        100       110       120

     120       130       140       150       160       170
Bari1   CTATGCAGAGTCAGATGAAAGAAGAATTGAAAAAATAACTGTTCCTATGCGCAAGGAAGA
        ::::::::::::::::::::::::::::::::::::::::::::::::::::::::::::
dimer   CTATGCAGAGTCAGATGAAAGAAGAATTGAAAAAATAACTGTTCCTATGCGCAAGGAAGA
               130       140       150       160       170       180

     180       190       200       210       220       230
Bari1   GGCAAATGAAGAGATCTTTATCAGTTGTCAGAAGTATTTGCACACGGTTTCGTCGCATCA
        ::::::::::::::::::::::::::::::::::::::::::::::::::::::::::::
dimer   GGCAAATGAAGAGATCTTTATCAGTTGTCAGAAGTATTTGCACACGGTTTCGTCGCATCA
               190       200       210       220       230       240

     240       250       260       270       280       290
Bari1   CAATTATTTTCACAACGCAATTTCTTCTTCAGTGATTGGTTTAGAGTGACAAGTGCCGGT
        ::::::::::::::::::::::::::::::::::::::::::::::::::::::::::::
dimer   CAATTATTTTCACAACGCAATTTCTTCTTCAGTGATTGGTTTAGAGTGACAAGTGCCGGT
               250       260       270       280       290       300

     300       310       320       330       340       350
Bari1   TTGTTTGCTTAAATACATTTAAATTATTGAATAAAAATTAGATTTAATCATTTTCCTATT
        ::::::::::::::::::::::::::::::::::::::::::::::::::::::::::::
dimer   TTGTTTGCTTAAATACATTTAAATTATTGAATAAAAATTAGATTTAATCATTTTCCTATT
               310       320       330       340       350       360

     360       370       380       390       400       410
Bari1   ACAGTTATTAAATAAAATGCCCAAAACAAAAGAGTTAACAGTTGAGGCCCGGGCTGGTAT
        ::::::::::::::::::::::::::::::::::::::::::::::::::::::::::::
dimer   ACAGTTATTAAATAAAATGCCCAAAACAAAAGAGTTAACAGTTGAGGCCCGGGCTGGTAT
               370       380       390       400       410       420

     420       430       440       450       460       470
Bari1   TGTTGCTAGGTTTAAAGCCGGTACACCTGCGGCCAAAATAGCTGAAATATATCAAATTTC
        ::::::::::::::::::::::::::::::::::::::::::::::::::::::::::::
dimer   TGTTGCTAGGTTTAAAGCCGGTACACCTGCGGCCAAAATAGCTGAAATATATCAAATTTC
               430       440       450       460       470       480

     480       490       500       510       520       530
Bari1   GCGTAGAACTGTCTACTACTTAATAAAAAAGTTTGATACAGTTGGCACATTAAAAAAATAA
        ::::::::::::::::::::::::::::::::::::::::::::::::::::::::::::
dimer   GCGTAGAACTGTCTACTACTTAATAAAAAAGTTTGATACAGTTGGCACATTAAAAAAATAA
               490       500       510       520       530       540

     540       550       560       570       580       590
Bari1   AAAAAGATCAGGCCGAAAACCTGTGCTGGACCAAAGGCAATGCAGGCAAATACTTGGAGT
        ::::::::::::::::::::::::::::::::::::::::::::::::::::::::::::
dimer   AAAAAGATCAGGCCGAAAACCTGTGCTGGACCAAAGGCAATGCAGGCAAATACTTGGAGT
               550       560       570       580       590       600

     600       610       620       630       640       650
Bari1   TGTGGCGAAGAATCCTAGTGCCAGTCCGGTAAAAATTGCCTTAGAATCAAAAAATACAAT
        ::::::::::::::::::::::::::::::::::::::::::::::::::::::::::::
dimer   TGTGGCGAAGAATCCTAGTGCCAGTCCGGTAAAAATTGCCTTAGAATCAAAAAATACAAT
               610       620       630       640       650       660

     660       670       680       690       700       710
Bari1   TGGCAAACAAGTTAGTAGTTCTACAATTCGTCGCAGGCTAAAAGAAGCTGATTTTAAGAC
        ::::::::::::::::::::::::::::::::::::::::::::::::::::::::::: 
dimer   TGGCAAACAAGTTAGTAGTTCTACAATTCGTCGCAGGCTAAAAGAAGCTGATTTTAAGAA
```

```
              670       680       690       700       710       720

     720       730       740       750       760       770
Bari1  ATACGTTGTTCGCAAAACGATTGAGATCACACCAACCAACAAAACAAAACGTCTTCGATT
       ::::::::::::::::::::::::::::::::::::::::::::::::::::::::::::
dimer  ATACGTTGTTCGCAAAACGATTGAGATCACACCAACCAACAAAACAAAACGTCTTCGATT
              730       740       750       760       770       780

     780       790       800       810       820       830
Bari1  TGCGTTGGAATATGTTAAGAAGCCTCTTGACTTTTGGTTTAATATTTTATGGACTGATGA
       ::::::::::::::::::::::::::::::::::::::::::::::::::::::::::::
dimer  TGCGTTGGAATATGTTAAGAAGCCTCTTGACTTTTGGTTTAATATTTTATGGACTGATGA
              790       800       810       820       830       840

     840       850       860       870       880       890
Bari1  GTCTGCATTTCAGTACCAGGGGTCATACAGCAAGCATTTTATGCATTTGAAAAATAATCA
       ::::::::::::::::::::::::::::::::::::::::::::::::::::::::::::
dimer  GTCTGCATTTCAGTACCAGGGGTCATACAGCAAGCATTTTATGCATTTGAAAAATAATCA
              850       860       870       880       890       900

     900       910       920       930       940       950
Bari1  AAAGCATTTGGCAGCCCAGCCAACCAATAGATTTGGTGGGGGCACAGTCATGTTTTGGGG
       ::::::::::::::::::::::::::::::::::::::::::::::::::::::::::::
dimer  AAAGCATTTGGCAGCCCAGCCAACCAATAGATTTGGTGGGGGCACAGTCATGTTTTGGGG
              910       920       930       940       950       960

     960       970       980       990      1000      1010
Bari1  ATGTCTTTCCTATTATGGATTCGGAGACTTGGTACCGATAGAAGGAACTTTAAATCAGAA
       ::::::::::::::::::::::::::::::::::::::::::::::::::::::::::::
dimer  ATGTCTTTCCTATTATGGATTCGGAGACTTGGTACCGATAGAAGGAACTTTAAATCAGAA
              970       980       990      1000      1010      1020

    1020      1030      1040      1050      1060      1070
Bari1  CGGATACCTTCTTATCTTAAACAACCATGCTTTTACGTCTGGAAATAGACTTTTTCCAAC
       ::::::::::::::::::::::::::::::::::::::::::::::::::::::::::::
dimer  CGGATACCTTCTTATCTTAAACAACCATGCTTTTACGTCTGGAAATAGACTTTTTCCAAC
             1030      1040      1050      1060      1070      1080

    1080      1090      1100      1110      1120      1130
Bari1  TACTGAATGGATTCTTCAGCAGGACAATGCTCCATGCCATAAGGGTAGGATACCAACAAA
       ::::::::::::::::::::::::::::::::::::::::::::::::::::::::::::
dimer  TACTGAATGGATTCTTCAGCAGGACAATGCTCCATGCCATAAGGGTAGGATACCAACAAA
             1090      1100      1110      1120      1130      1140

    1140      1150      1160      1170      1180      1190
Bari1  ATTTTTAAACGACCTTAATCTGGCGGTTCTTCCGTGGCCCCCCCAAAGCCCAGACCTTAA
       ::::::::::::::::::::::::::::::::::::::::::::::::::::::::::::
dimer  ATTTTTAAACGACCTTAATCTGGCGGTTCTTCCGTGGCCCCCCCAAAGCCCAGACCTTAA
             1150      1160      1170      1180      1190      1200

    1200      1210      1220      1230      1240      1250
Bari1  TATCATTGAAAACGTTTGGGCTTTTATTAAAAACCAACGAACTATTGATAAAAATAGAAA
       ::::::::::::::::::::::::::::::::::::::::::::::::::::::::::::
dimer  TATCATTGAAAACGTTTGGGCTTTTATTAAAAACCAACGAACTATTGATAAAAATAGAAA
             1210      1220      1230      1240      1250      1260

    1260      1270      1280      1290      1300      1310
Bari1  ACGAGAGGGAGCCATCATTGAAATAGCGGAGATTTGGTCCAAATTGACATTAGAATTTGC
       ::::::::::::::::::::::::::::::::::::::::::::::::::::::::::::
dimer  ACGAGAGGGAGCCATCATTGAAATAGCGGAGATTTGGTCCAAATTGACATTAGAATTTGC
             1270      1280      1290      1300      1310      1320

    1320      1330      1340      1350      1360      1370
Bari1  ACAAACTTTGGTAAGGTCAATACCAAAAAGACTTCAAGCAGTTATTGATGCCAAAGGTGG
       ::::::::::::::::::::::::::::::::::::::::::::::::::::::::::::
dimer  ACAAACTTTGGTAAGGTCAATACCAAAAAGACTTCAAGCAGTTATTGATGCCAAAGGTGG
             1330      1340      1350      1360      1370      1380

    1380      1390      1400      1410      1420      1430
Bari1  TGTTACAAAATATTAGTATTGTATTTATATAAAATAAAGAAATTCTTATGTTGAAATTAG
       :::::::::::::::::::::::::::::::::::::::::::::::::::: :::::::
dimer  TGTTACAAAATATTAGTATTGTATTTATATAAAATAAAGAAATTCTTATGTTTAAATTAG
             1390      1400      1410      1420      1430      1440

    1440      1450      1460      1470      1480      1490
```

```
Bari1  ATGTTAAGCTGAAATTTACTAAATTAAGTTGAGTGAAAATACTTTTGAAGCGCAATAAAC
       :::::::::::::::::::::::::::::::::::::::
dimer  ATGTTAAGCTGAAATTTACTAAATTAAGTTGAGTGAAAA---------------------
             1450      1460      1470

    1500      1510      1520      1530      1540      1550
Bari1  ATGTGAAAATACTATTGACAACTTGCATGCATATTTTCTTTTGCTTTAAGCTTTGTACTA

dimer  ------------------------------------------------------------


    1560      1570      1580      1590      1600      1610
Bari1  TGAACCGTTATCTTTCGTATTTCTTTTCGACTACCTTCTGCATAGATCAAGCTAAGCGAT

dimer  ------------------------------------------------------------


    1620      1630      1640      1650      1660      1670
Bari1  AAGAACTATTTCAGGCAAATCGGACAACAACAAGAAGAAATATAACAAAAAGAAGTTGAA

dimer  ------------------------------------------------------------


    1680      1690      1700      1710      1720      1730
Bari1  GTTTGCAAATATTGTGCGTTGTGAAAATACTTTTGACCACCTCTGGTCATGGTCAAAATT

dimer  ------------------------------------------------------------


    1740      1750      1760      1770      1780      1790
Bari1  ATTTTCACAAAGTGCATTTTTGTGCATGGGTCACAAACAGTTGCTTGTGCAGCAAGTGGG

dimer  ------------------------------------------------------------


    1800      1810      1820      1830      1840      1850
Bari1  GGGAGGTGAAATGCAAAAAAACTTTTGCTTTTGCAAATTCAAACCTATGCAGAGTCAGAT
                          :::::::::::::::::::::::::::::::::::::::::
dimer  -------------------AACTTTTGCTTTTGCAAATTCAAACCTATGCAGAGTCAGAT
                       1480      1490      1500      1510      1520

    1860      1870      1880      1890      1900      1910
Bari1  GAAAGAAGAATTGAAAAAATAACTGTTCCTATGCGCAAGGAAGAGGCAAATGAAGAGATC
       ::::::::::::::::::::::::::::::::::::::::::::::::::::::::::::
dimer  GAAAGAAGAATTGAAAAAATAACTGTTCCTATGCGCAAGGAAGAGGCAAATGAAGAGATC
             1530      1540      1550      1560      1570      1580

    1920      1930      1940      1950      1960      1970
Bari1  TTTATCAGTTGTCAGAAGTATTTGCACACGGTTTCGTCGCATCACAATTATTTTCACAAC
       ::::::::::::::::::::::::::::::::::::::::::::::::::::::::::::
dimer  TTTATCAGTTGTCAGAAGTATTTGCACACGGTTTCGTCGCATCACAATTATTTTCACAAC
             1590      1600      1610      1620      1630      1640

    1980      1990      2000      2010      2020      2030
Bari1  GCAATTTCTTCTTCAGTGATTGGTTTAGAGTGACAAGTGCCGGTTTGTTTGCTTAAATAC
       ::::::::::::::::::::::::::::::::::::::::::::::::::::::::::::
dimer  GCAATTTCTTCTTCAGTGATTGGTTTAGAGTGACAAGTGCCGGTTTGTTTGCTTAAATAC
             1650      1660      1670      1680      1690      1700

    2040      2050      2060      2070      2080      2090
Bari1  ATTTAAATTATTGAATAAAAATTAGATTTAATCATTTTCCTATTACAGTTATTAAATAAA
       ::::::::::::::::::::::::::::::::::::::::::::::::::::::::::::
dimer  ATTTAAATTATTGAATAAAAATTAGATTTAATCATTTTCCTATTACAGTTATTAAATAAA
             1710      1720      1730      1740      1750      1760

    2100      2110      2120      2130      2140      2150
Bari1  ATGCCCAAAACAAAAGAGTTAACAGTTGAGGCCCGGGCTGGTATTGTTGCTAGGTTTAAA
       ::::::::::::::::::::::::::::::::::::::::::::::::::::::::::::
dimer  ATGCCCAAAACAAAAGAGTTAACAGTTGAGGCCCGGGCTGGTATTGTTGCTAGGTTTAAA
             1770      1780      1790      1800      1810      1820

    2160      2170      2180      2190      2200      2210
Bari1  GCCGGTACACCTGCGGCCAAAATAGCTGAAATATATCAAATTTCGCGTAGAACTGTCTAC
       ::::::::::::::::::::::::::::::::::::::::::::::::::::::::::::
dimer  GCCGGTACACCTGCGGCCAAAATAGCTGAAATATATCAAATTTCGCGTAGAACTGTCTAC
```

```
              1830      1840      1850      1860      1870      1880

     2220      2230      2240      2250      2260      2270
Bari1  TACTTAATAAAAAAGTTTGATACAGTTGGCACATTAAAAAATAAAAAAAGATCAGGCCGA
       ::::::::::::::::::::::::::::::::::::::::::::::::::::::::::::
dimer  TACTTAATAAAAAAGTTTGATACAGTTGGCACATTAAAAAATAAAAAAAGATCAGGCCGA
             1890      1900      1910      1920      1930      1940

     2280      2290      2300      2310      2320      2330
Bari1  AAACCTGTGCTGGACCAAAGGCAATGCAGGCAAATACTTGGAGTTGTGGCGAAGAATCCT
       ::::::::::::::::::::::::::::::::::::::::::::::::::::::::::::
dimer  AAACCTGTGCTGGACCAAAGGCAATGCAGGCAAATACTTGGAGTTGTGGCGAAGAATCCT
             1950      1960      1970      1980      1990      2000

     2340      2350      2360      2370      2380      2390
Bari1  AGTGCCAGTCCGGTAAAAATTGCCTTAGAATCAAAAAATACAATTGGCAAACAAGTTAGT
       ::::::::::::::::::::::::::::::::::::::::::::::::::::::::::::
dimer  AGTGCCAGTCCGGTAAAAATTGCCTTAGAATCAAAAAATACAATTGGCAAACAAGTTAGT
             2010      2020      2030      2040      2050      2060

     2400      2410      2420      2430      2440      2450
Bari1  AGTTCTACAATTCGTCGCAGGCTAAAAGAAGCTGATTTTAAGACATACGTTGTTCGCAAA
       ::::::::::::::::::::::::::::::::::::::::::::::::::::::::::::
dimer  AGTTCTACAATTCGTCGCAGGCTAAAAGAAGCTGATTTTAAGACATACGTTGTTCGCAAA
             2070      2080      2090      2100      2110      2120

     2460      2470      2480      2490      2500      2510
Bari1  ACGATTGAGATCACACCAACCAACAAAACAAAACGTCTTCGATTTGCGTTGGAATATGTT
       ::::::::::::::::::::::::::::::::::::::::::::::::::::::::::::
dimer  ACGATTGAGATCACACCAACCAACAAAACAAAACGTCTTCGATTTGCGTTGGAATATGTT
             2130      2140      2150      2160      2170      2180

     2520      2530      2540      2550      2560      2570
Bari1  AAGAAGCCTCTTGACTTTTGGTTTAATATTTTATGGACTGATGAGTCTGCATTTCAGTAC
       ::::::::::::::::::::::::::::::::::::::::::::::::::::::::::::
dimer  AAGAAGCCTCTTGACTTTTGGTTTAATATTTTATGGACTGATGAGTCTGCATTTCAGTAC
             2190      2200      2210      2220      2230      2240

     2580      2590      2600      2610      2620      2630
Bari1  CAGGGGTCATACAGCAAGCATTTTATGCATTTGAAAAATAATCAAAAGCATTTGGCAGCC
       ::::::::::::::::::::::::::::::::::::::::::::::::::::::::::::
dimer  CAGGGGTCATACAGCAAGCATTTTATGCATTTGAAAAATAATCAAAAGCATTTGGCAGCC
             2250      2260      2270      2280      2290      2300

     2640      2650      2660      2670      2680      2690
Bari1  CAGCCAACCAATAGATTTGGTGGGGGCACAGTCATGTTTTGGGGATGTCTTTCCTATTAT
       ::::::::::::::::::::::::::::::::::::::::::::::::::::::::::::
dimer  CAGCCAACCAATAGATTTGGTGGGGGCACAGTCATGTTTTGGGGATGTCTTTCCTATTAT
             2310      2320      2330      2340      2350      2360

     2700      2710      2720      2730      2740      2750
Bari1  GGATTCGGAGACTTGGTACCGATAGAAGGAACTTTAAATCAGAACGGATACCTTCTTATC
       ::::::::::::::::::::::::::::::::::::::::::::::::::::::::::::
dimer  GGATTCGGAGACTTGGTACCGATAGAAGGAACTTTAAATCAGAACGGATACCTTCTTATC
             2370      2380      2390      2400      2410      2420

     2760      2770      2780      2790      2800      2810
Bari1  TTAAACAACCATGCTTTTACGTCTGGAAATAGACTTTTTCCAACTACTGAATGGATTCTT
       ::::::::::::::::::::::::::::::::::::::::::::::::::::::::::::
dimer  TTAAACAACCATGCTTTTACGTCTGGAAATAGACTTTTTCCAACTACTGAATGGATTCTT
             2430      2440      2450      2460      2470      2480

     2820      2830      2840      2850      2860      2870
Bari1  CAGCAGGACAATGCTCCATGCCATAAGGGTAGGATACCAACAAAATTTTTAAACGACCTT
       ::::::::::::::::::::::::::::::::::::::::::::::::::::::::::::
dimer  CAGCAGGACAATGCTCCATGCCATAAGGGTAGGATACCAACAAAATTTTTAAACGACCTT
             2490      2500      2510      2520      2530      2540

     2880      2890      2900      2910      2920      2930
Bari1  AATCTGGCGGTTCTTCCGTGGCCCCCCCAAAGCCCAGACCTTAATATCATTGAAAACGTT
       ::::::::::::::::::::::::::::::::::::::::::::::::::::::::::::
dimer  AATCTGGCGGTTCTTCCGTGGCCCCCCCAAAGCCCAGACCTTAATATCATTGAAAACGTT
             2550      2560      2570      2580      2590      2600

     2940      2950      2960      2970      2980      2990
```

```
Bari1  TGGGCTTTTATTAAAAACCAACGAACTATTGATAAAAATAGAAAACGAGAGGGAGCCATC
       ::::::::::::::::::::::::::::::::::::::::::::::::::: ::::::::
dimer  TGGGCTTTTATTAAAAACCAACGAACTATTGATAAAAATAGAAAACGAGAGTGAGCCATC
             2610      2620      2630      2640      2650      2660

    3000      3010      3020      3030      3040      3050
Bari1  ATTGAAATAGCGGAGATTTGGTCCAAATTGACATTAGAATTTGCACAAACTTTGGTAAGG
       ::::::::::::::::::::::::::::::::::::::::::::::::::::::::::::
dimer  ATTGAAATAGCGGAGATTTGGTCCAAATTGACATTAGAATTTGCACAAACTTTGGTAAGG
             2670      2680      2690      2700      2710      2720

    3060      3070      3080      3090      3100      3110
Bari1  TCAATACCAAAAAGACTTCAAGCAGTTATTGATGCCAAAGGTGGTGTTACAAAATATTAG
       ::::::::::::::::::::::::::::::::::::::::::::::::::::::::::::
dimer  TCAATACCAAAAAGACTTCAAGCAGTTATTGATGCCAAAGGTGGTGTTACAAAATATTAG
             2730      2740      2750      2760      2770      2780

    3120      3130      3140      3150      3160      3170
Bari1  TATTGTATTTATATAAAATAAAGAAATTCTTATGTTGAAATTAGATGTTAAGCTGAAATT
       ::::::::::::::::::::::::::::::::::::::::::::::::::::::::::::
dimer  TATTGTATTTATATAAAATAAAGAAATTCTTATGTTGAAATTAGATGTTAAGCTGAAATT
             2790      2800      2810      2820      2830      2840

    3180      3190      3200      3210      3220      3230
Bari1  TACTAAATTAAGTTGAGTGAAAATACTTTTGAAGCGCAATAAACATGTGAAAATACTATT
       ::::::::::::::::::::::::::::::::::::::::::::::::::::::::::::
dimer  TACTAAATTAAGTTGAGTGAAAATACTTTTGAAGCGCAATAAACATGTGAAAATACTATT
             2850      2860      2870      2880      2890      2900

    3240      3250      3260      3270      3280      3290
Bari1  GACAACTTGCATGCATATTTTCTTTTGCTTTAAGCTTTGTACTATGAACCGTTATCTTTC
       ::::::::::::::::::::::::::::::::::::::::::::::::::::::::::::
dimer  GACAACTTGCATGCATATTTTCTTTTGCTTTAAGCTTTGTACTATGAACCGTTATCTTTC
             2910      2920      2930      2940      2950      2960

    3300      3310      3320      3330      3340      3350
Bari1  GTATTTCTTTTCGACTACCTTCTGCATAGATCAAGCTAAGCGATAAGAACTATTTCAGGC
       ::::::::::::::::::::::::::::::::::::::::::::::::::::::::::::
dimer  GTATTTCTTTTCGACTACCTTCTGCATAGATCAAGCTAAGCGATAAGAACTATTTCAGGC
             2970      2980      2990      3000      3010      3020

    3360      3370      3380      3390      3400      3410
Bari1  AAATCGGACAACAACAAGAAGAAATATAACAAAAAGAAGTTGAAGTTTGCAAATATTGTG
       ::::::::::::::::::::::::::::::::::::::::::::::::::::::::::::
dimer  AAATCGGACAACAACAAGAAGAAATATAACAAAAAGAAGTTGAAGTTTGCAAATATTGTG
             3030      3040      3050      3060      3070      3080

    3420      3430      3440
Bari1  CGTTGTGAAAATACTTTTGACCACCTCTG
       :::::::::::::::::::::::::::::
dimer  CGTTGTGAAAATACTTTTGACCACCTCTG
             3090      3100
```

global sequence alignment comparing the deleted elements in the scaffold utg7180000000502 with a canonical Bari1 heterochromatic dimer.

Homologous sequences involved in an intra-molecular recombination event are highlighted in red
