# Supplementary material for: Identification of Bari Transposons in 23 Sequenced Drosophila Genomes Reveals Novel Structural Variants, MITEs and Horizontal Transfer
Source: PLoS One. 2016 May 23;11(5):e0156014. doi: 10.1371/journal.pone.0156014 (PMC4877112; doi:10.1371/journal.pone.0156014)
Supplement: S5 Fig — The red-boxed area in each plot indicates area of significant (p<0,05) enrichment (positive values) or depletion (negative values) of the analyzed transposable elements’ super-families in the proximity of Bari elements in a sequence range of 2,5 kb. X axes report p value. Y axes report the distance from origin in bp. (PDF) [file pone.0156014.s005.pdf]

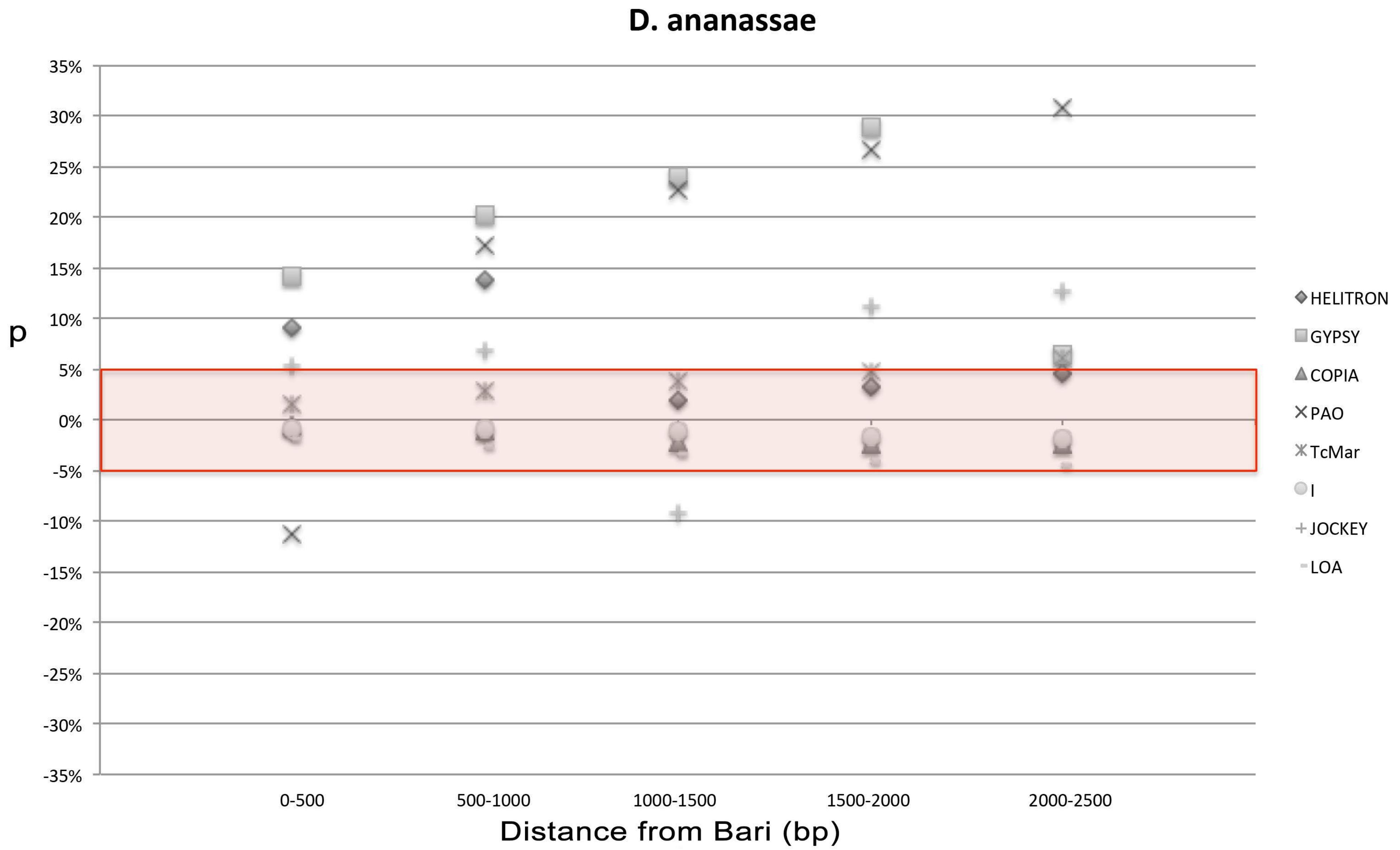
D. ananassae
35%
30%
25%
20%
15%
10%
5%
0%
-5%
-10%
-15%
-20%
-25%
-30%
-35%
p
0-500
500-1000
1000-1500
1500-2000
2000-2500
Distance from Bari (bp)
HELITRON
GYPSY
COPIA
PAO
TcMar
I
JOCKEY
LOA

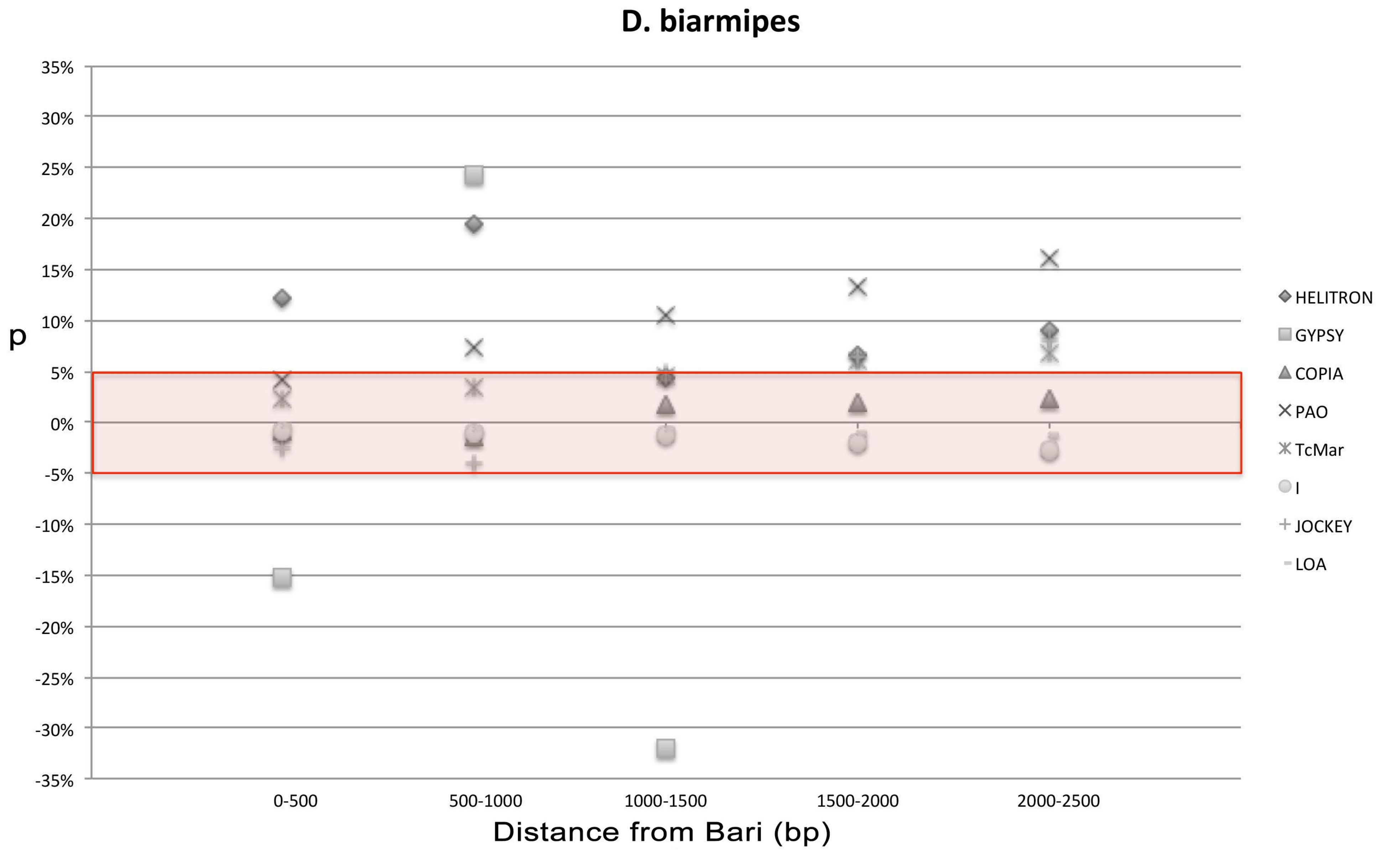
D. biarmipes
35%
30%
25%
20%
15%
10%
5%
0%
-5%
-10%
-15%
-20%
-25%
-30%
-35%
p
0-500
500-1000
1000-1500
1500-2000
2000-2500
Distance from Bari (bp)
HELITRON
GYPSY
COPIA
PAO
TcMar
I
JOCKEY
LOA

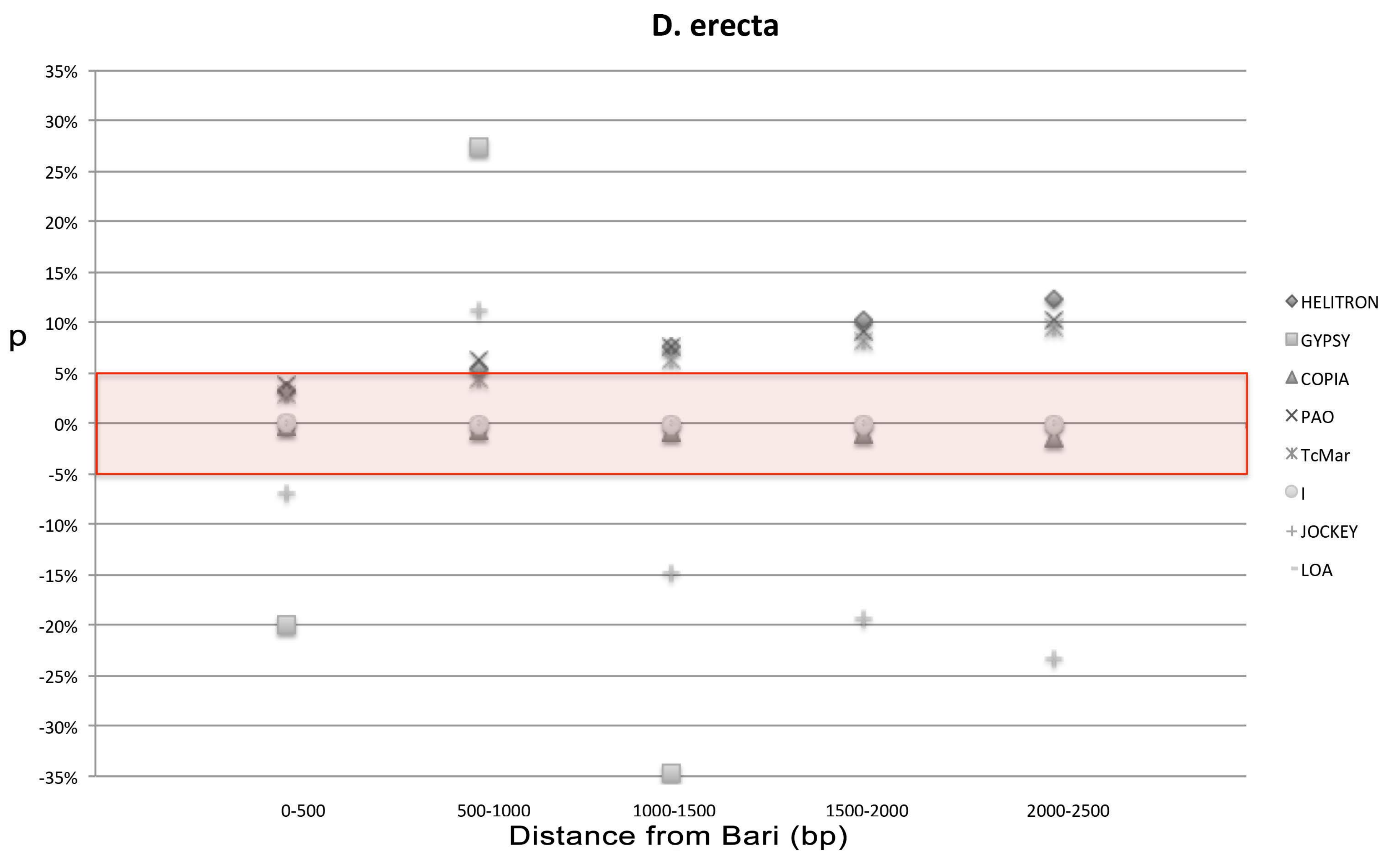
D. erecta
35%
30%
25%
20%
15%
10%
5%
0%
-5%
-10%
-15%
-20%
-25%
-30%
-35%
p
0-500
500-1000
1000-1500
1500-2000
2000-2500
Distance from Bari (bp)
HELITRON
GYPSY
COPIA
PAO
TcMar
I
JOCKEY
LOA

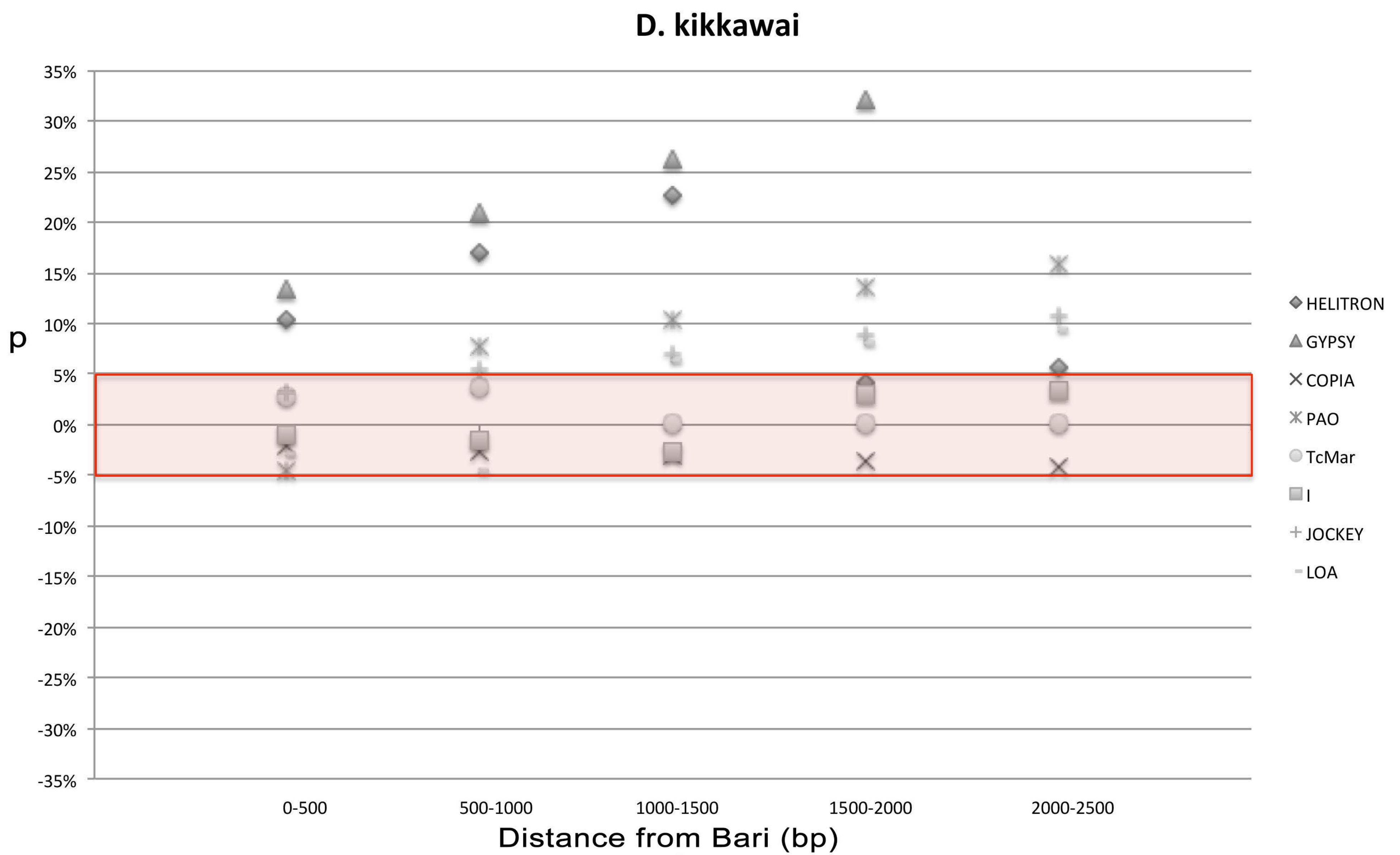

D. kikkawai
35%
30%
25%
20%
15%
10%
5%
0%
-5%
-10%
-15%
-20%
-25%
-30%
-35%
p
0-500
500-1000
1000-1500
1500-2000
2000-2500
Distance from Bari (bp)
HELITRON
GYPSY
COPIA
PAO
TcMar
I
JOCKEY
LOA

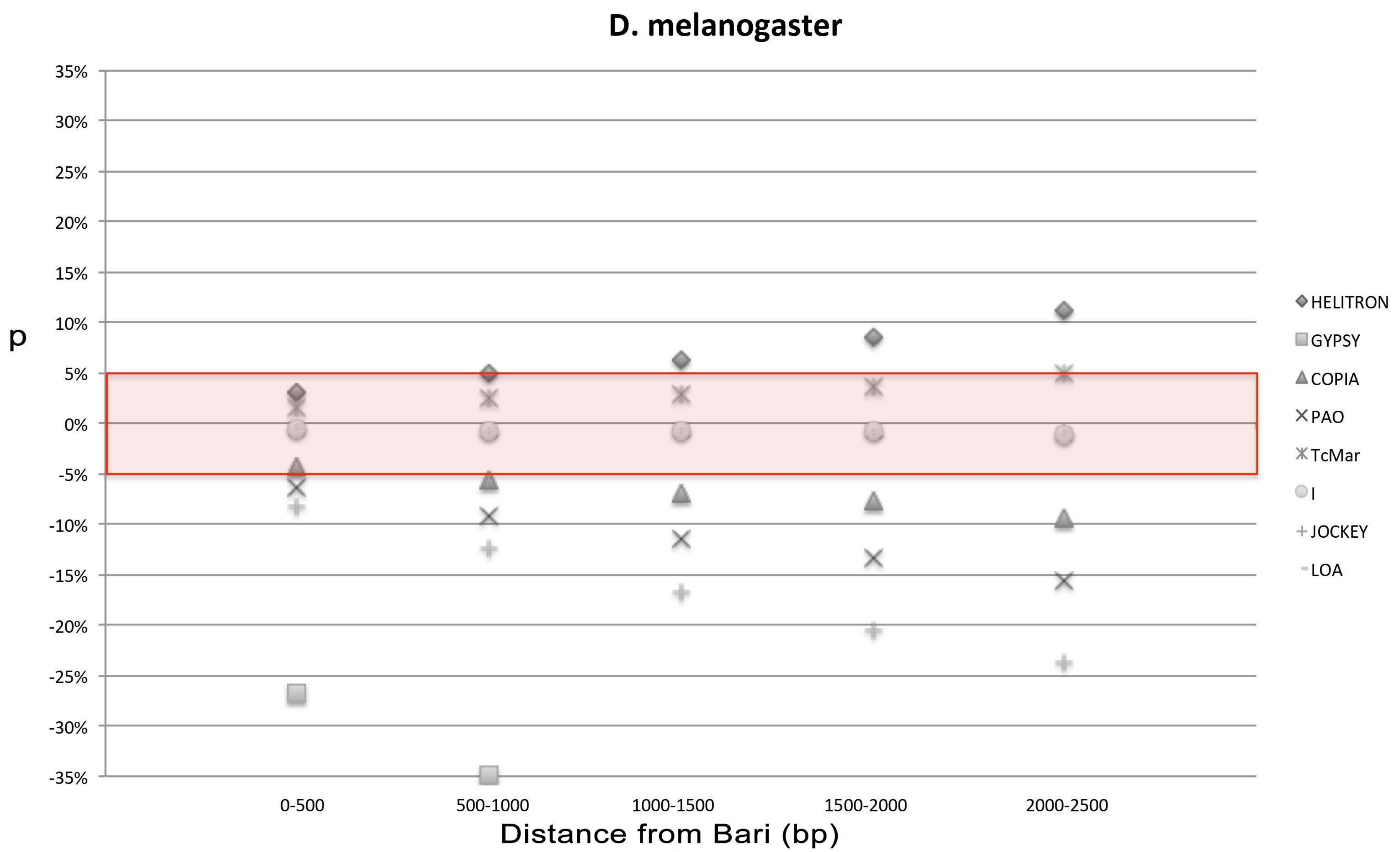
D. melanogaster
p
35%
30%
25%
20%
15%
10%
5%
0%
-5%
-10%
-15%
-20%
-25%
-30%
-35%
0-500
500-1000
1000-1500
1500-2000
2000-2500
Distance from Bari (bp)
HELITRON
GYPSY
COPIA
PAO
TcMar
I
JOCKEY
LOA

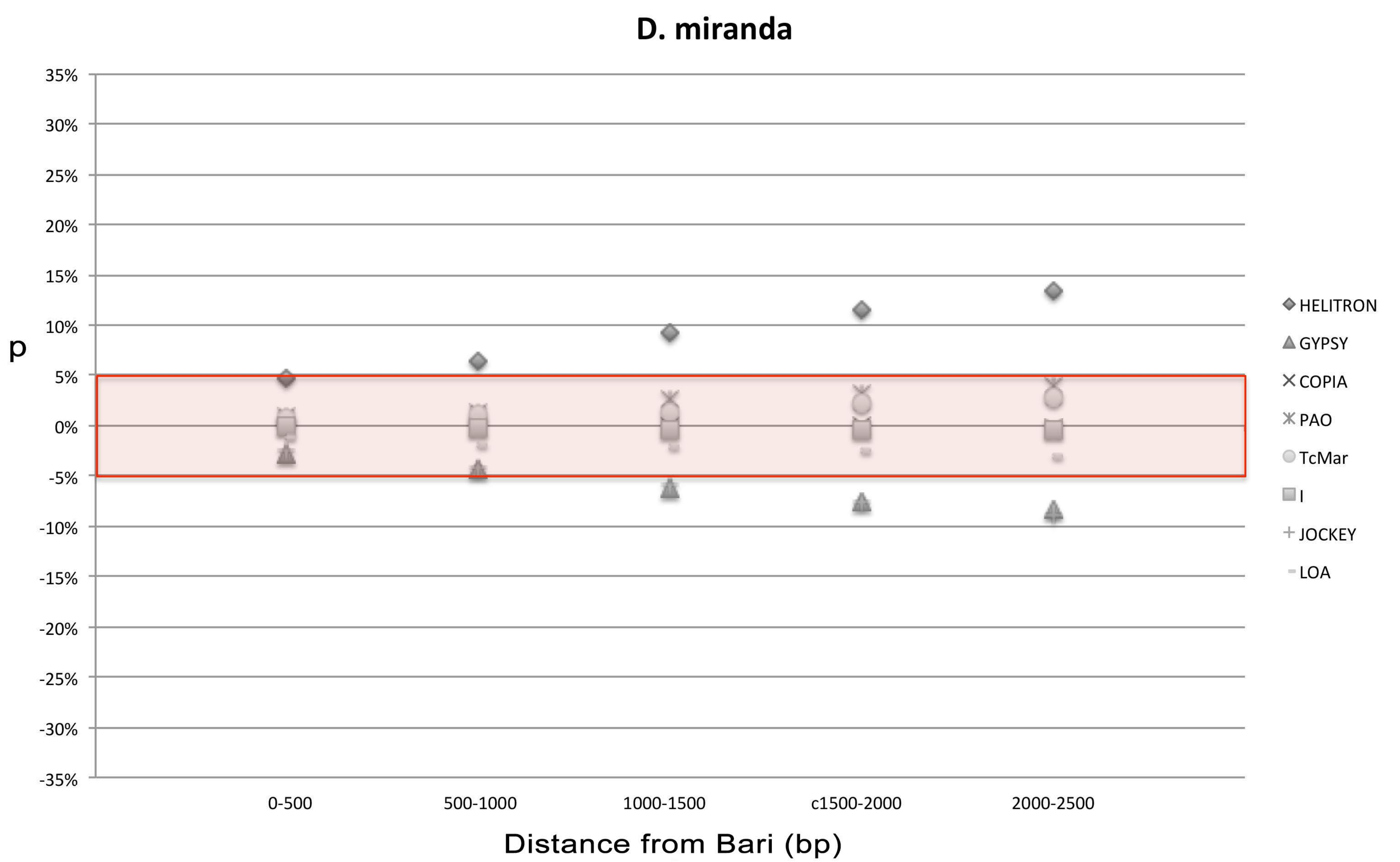
D. miranda
35%
30%
25%
20%
15%
10%
5%
0%
-5%
-10%
-15%
-20%
-25%
-30%
-35%
p
0-500
500-1000
1000-1500
c1500-2000
2000-2500
Distance from Bari (bp)
HELITRON
GYPSY
COPIA
PAO
TcMar
I
JOCKEY
LOA

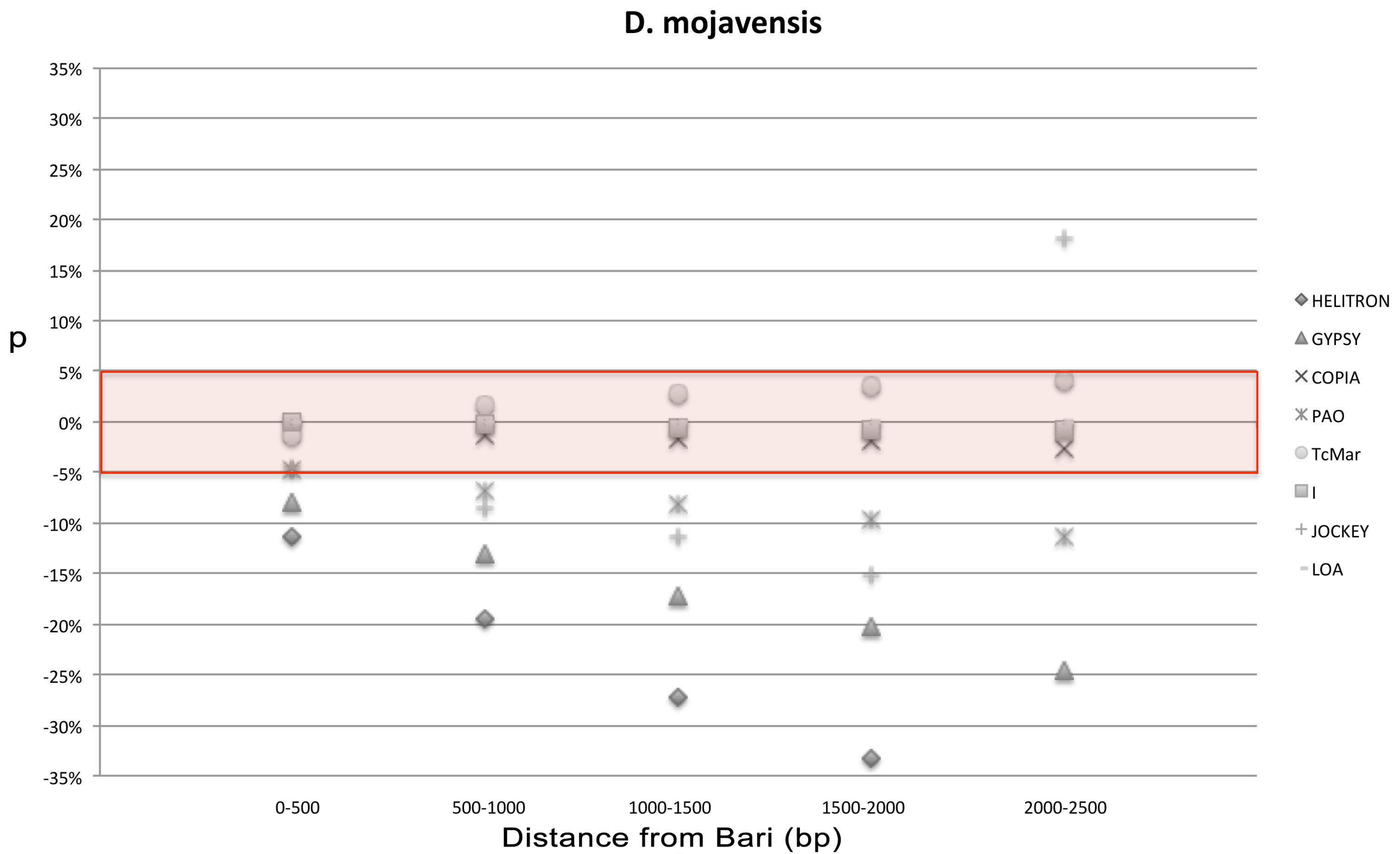
D. mojavensis
35%
30%
25%
20%
15%
10%
5%
0%
-5%
-10%
-15%
-20%
-25%
-30%
-35%
p
0-500
500-1000
1000-1500
1500-2000
2000-2500
Distance from Bari (bp)
HELITRON
GYPSY
COPIA
PAO
TcMar
I
JOCKEY
LOA

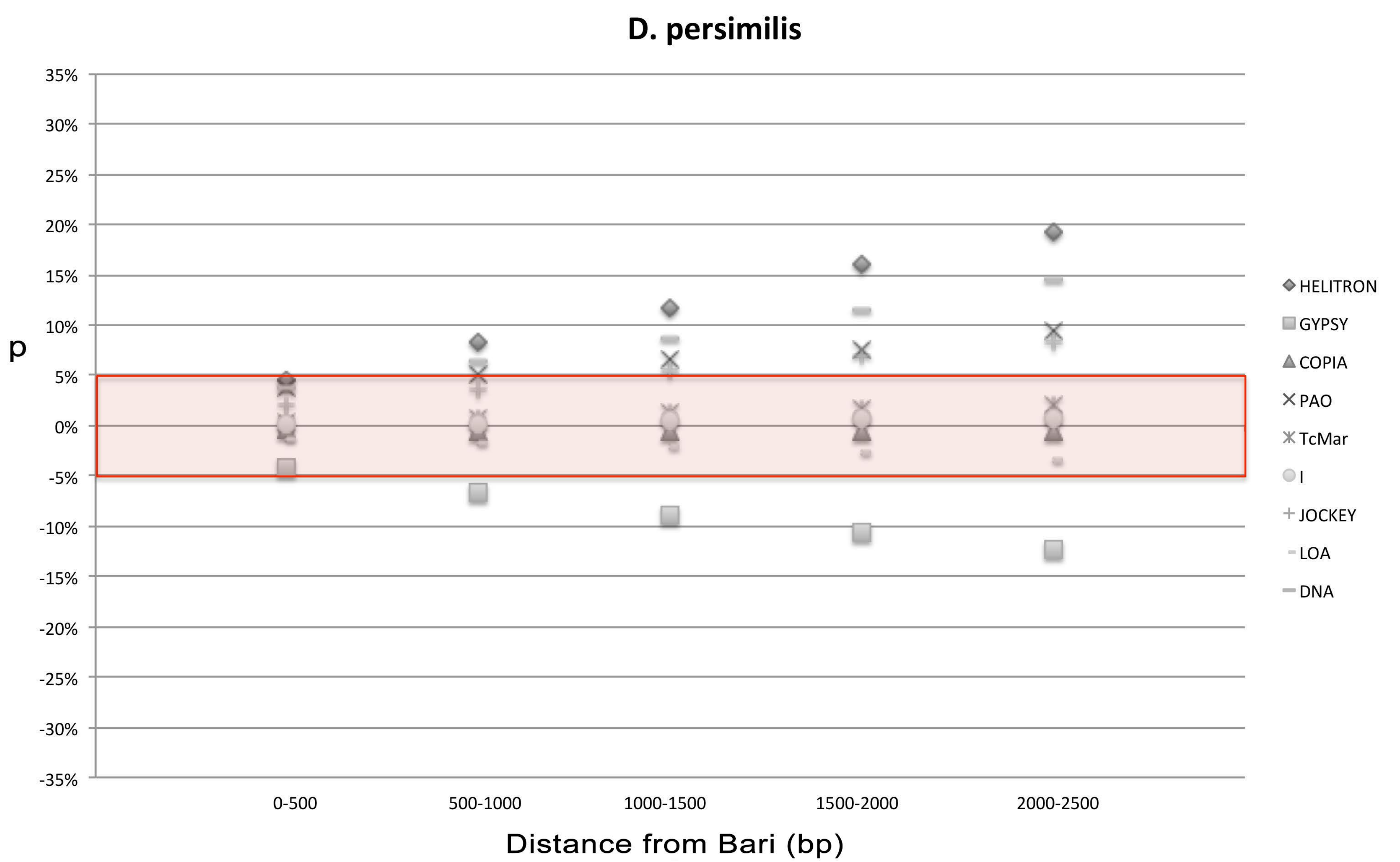
D. persimilis
p
35%
30%
25%
20%
15%
10%
5%
0%
-5%
-10%
-15%
-20%
-25%
-30%
-35%
0-500
500-1000
1000-1500
1500-2000
2000-2500
Distance from Bari (bp)
HELITRON
GYPSY
COPIA
PAO
TcMar
I
JOCKEY
LOA
DNA

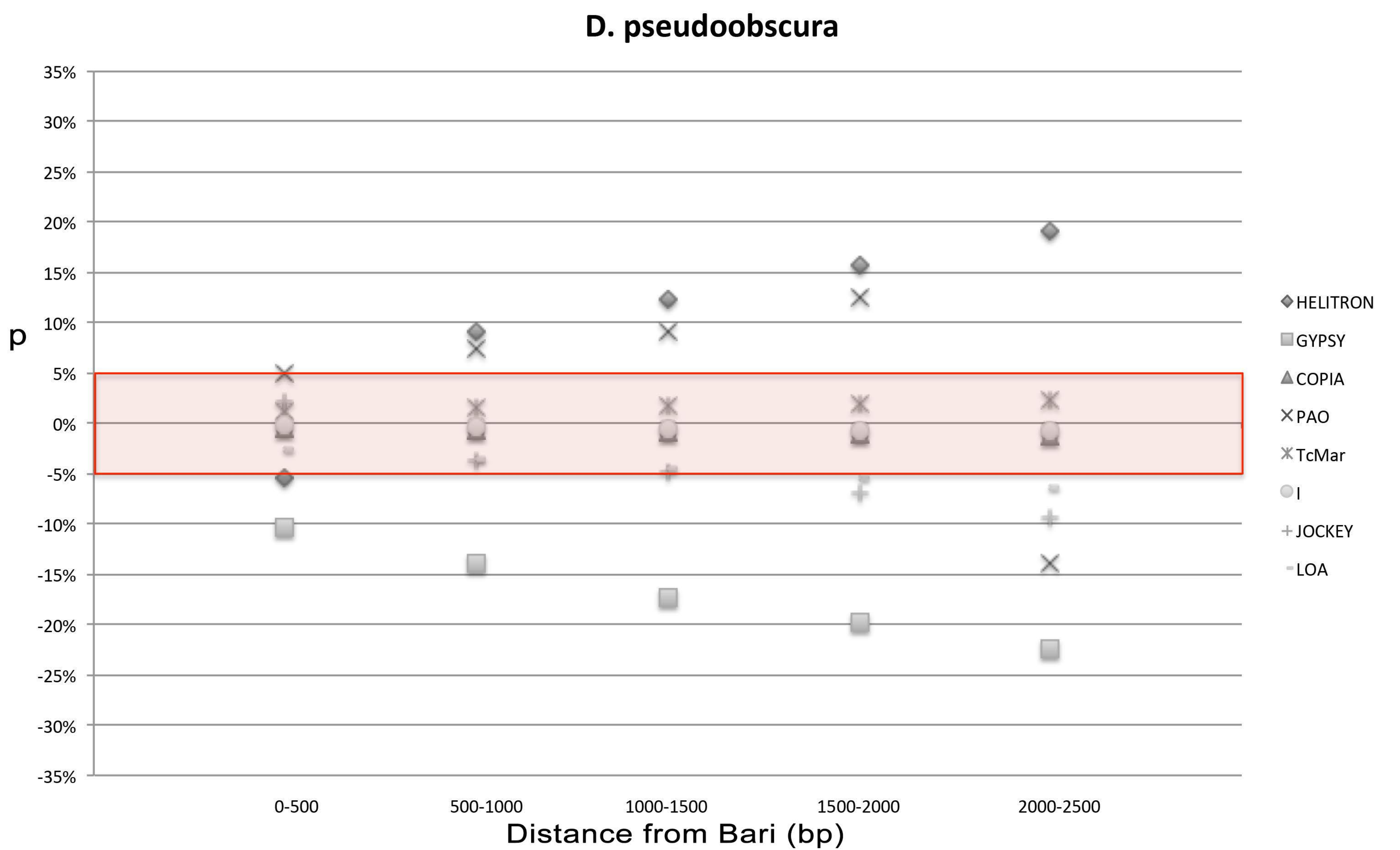
D. pseudoobscura
35%
30%
25%
20%
15%
10%
5%
0%
-5%
-10%
-15%
-20%
-25%
-30%
-35%
p
HELITRON
GYPSY
COPIA
PAO
TcMar
I
JOCKEY
LOA
0-500
500-1000
1000-1500
1500-2000
2000-2500
Distance from Bari (bp)

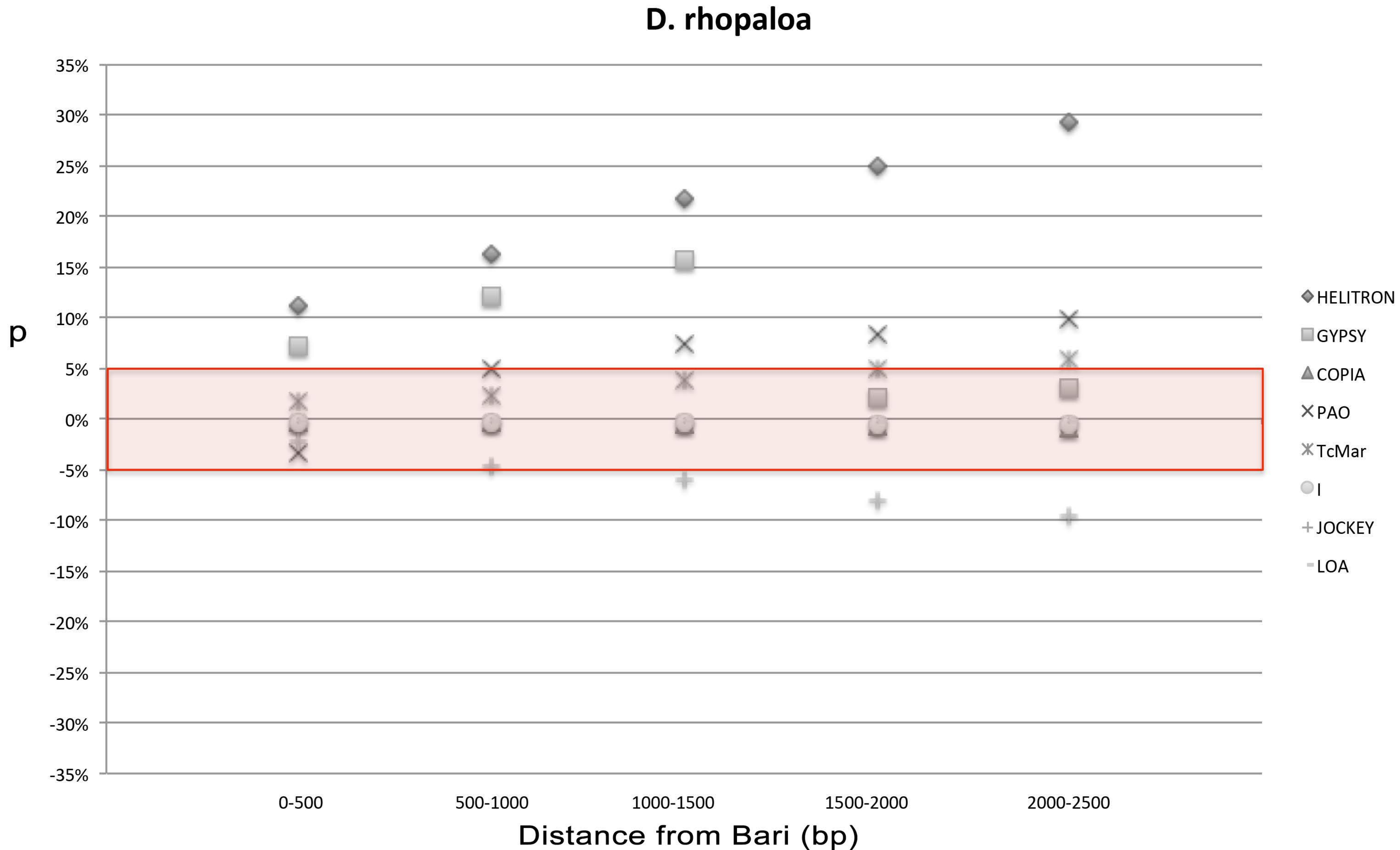

D. rhopaloa
35%
30%
25%
20%
15%
10%
5%
0%
-5%
-10%
-15%
-20%
-25%
-30%
-35%
p
0-500
500-1000
1000-1500
1500-2000
2000-2500
Distance from Bari (bp)
HELITRON
GYPSY
COPIA
PAO
TcMar
I
JOCKEY
LOA

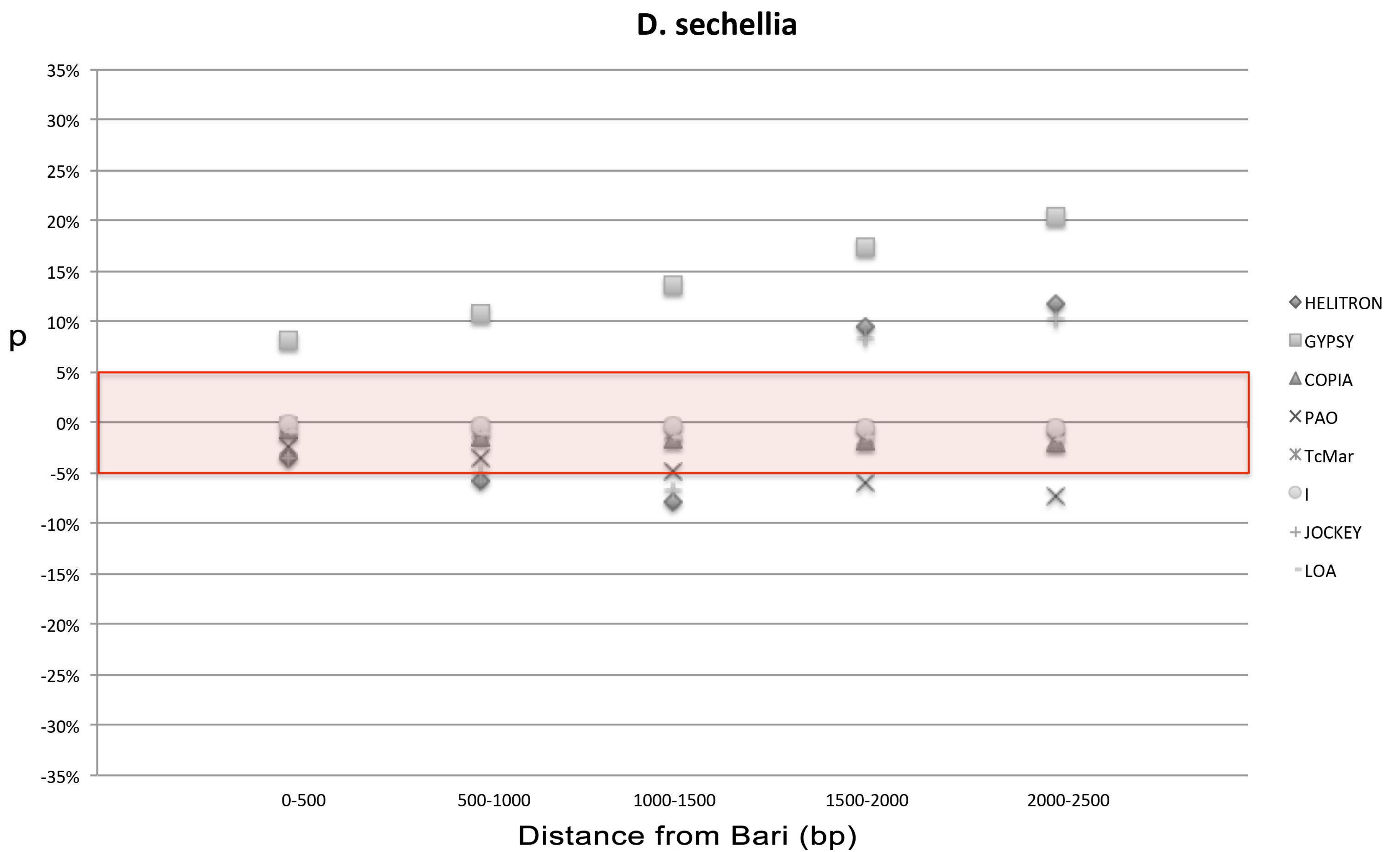
D. sechellia
35%
30%
25%
20%
15%
10%
5%
0%
-5%
-10%
-15%
-20%
-25%
-30%
-35%
p
0-500
500-1000
1000-1500
1500-2000
2000-2500
Distance from Bari (bp)
HELITRON
GYPSY
COPIA
PAO
TcMar
I
JOCKEY
LOA

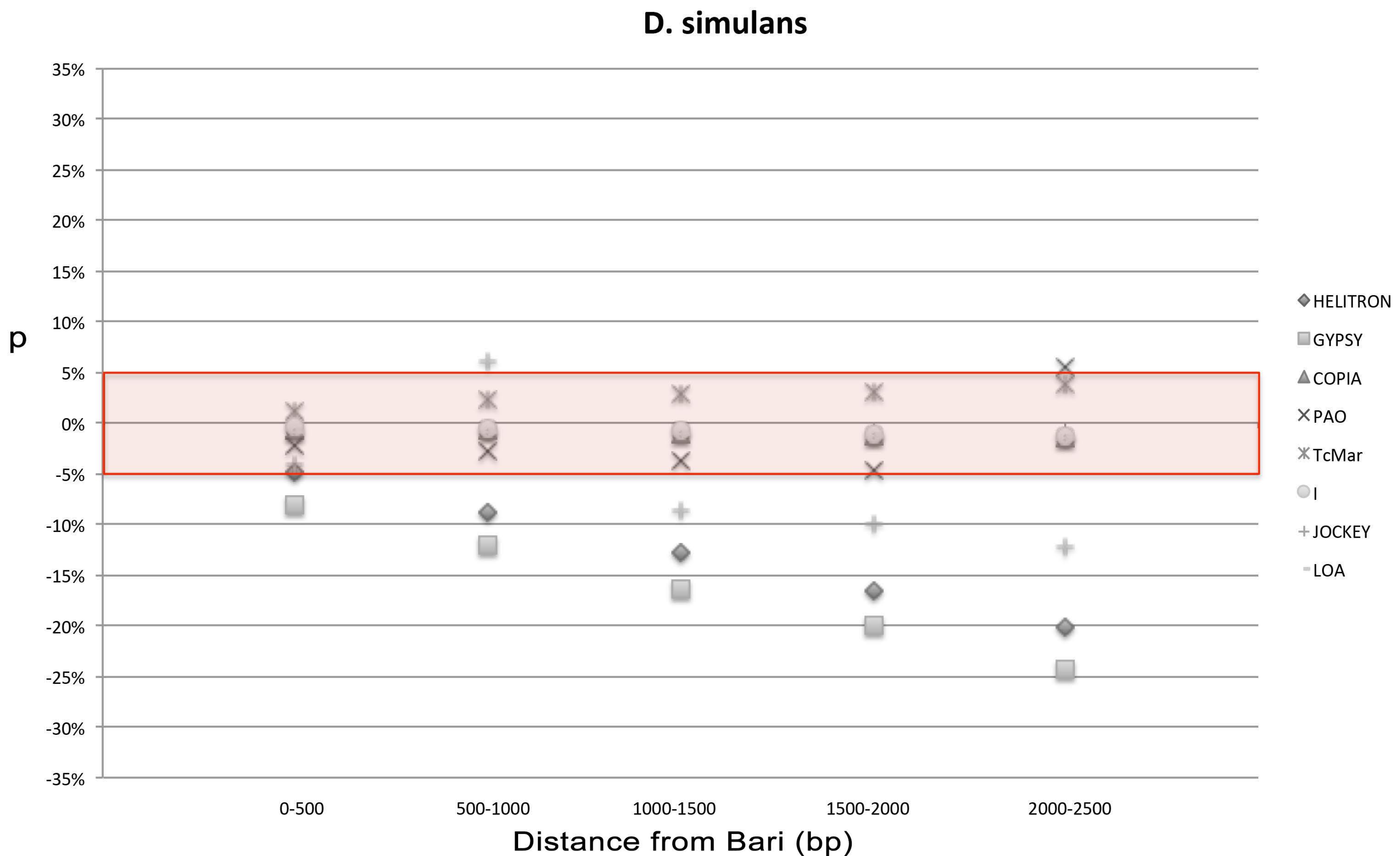
D. simulans
35%
30%
25%
20%
15%
10%
5%
0%
-5%
-10%
-15%
-20%
-25%
-30%
-35%
p
HELITRON
GYPSY
COPIA
PAO
TcMar
I
JOCKEY
LOA
0-500
500-1000
1000-1500
1500-2000
2000-2500
Distance from Bari (bp)

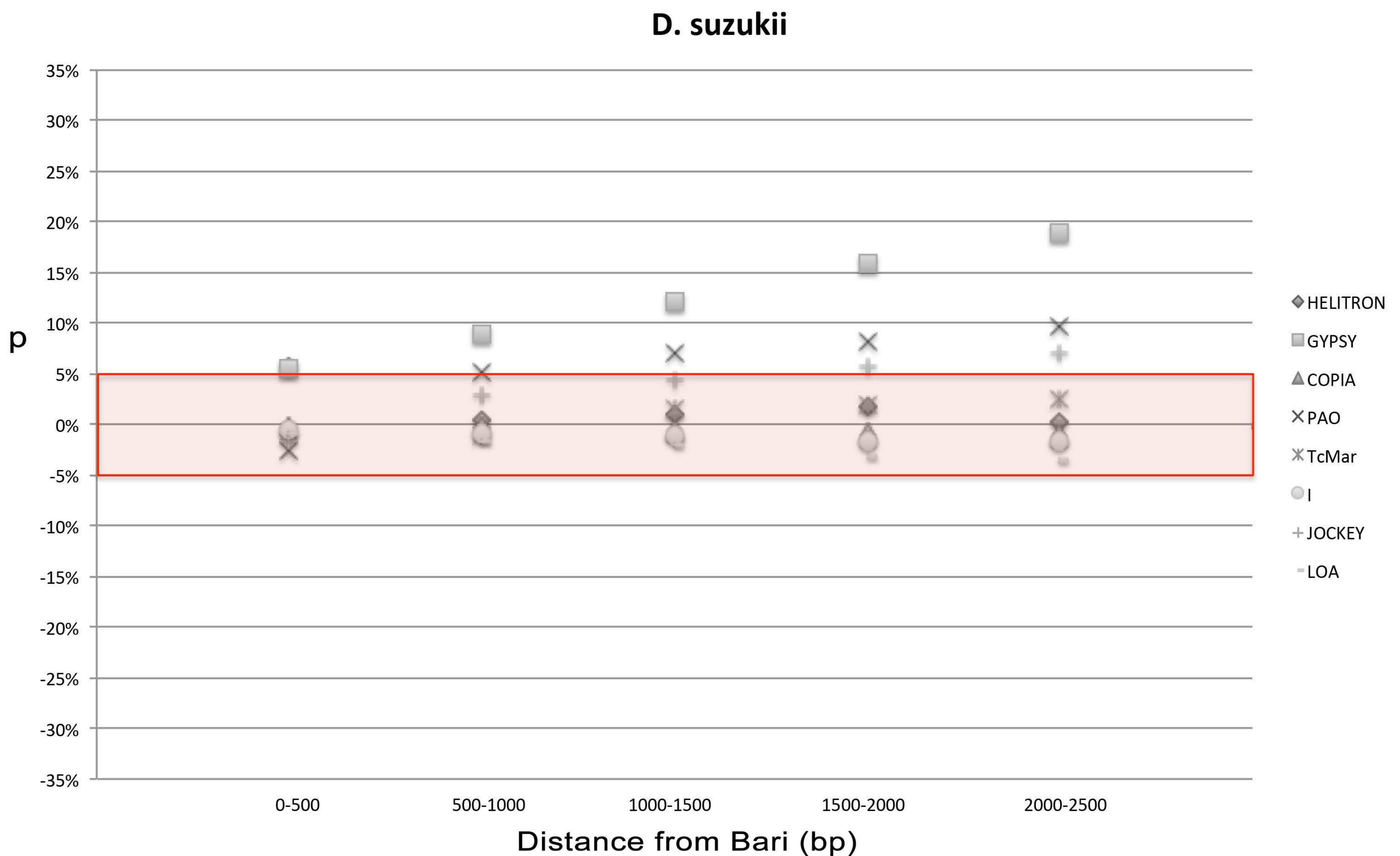
D. suzukii
35%
30%
25%
20%
15%
10%
5%
0%
-5%
-10%
-15%
-20%
-25%
-30%
-35%
p
0-500
500-1000
1000-1500
1500-2000
2000-2500
Distance from Bari (bp)
HELITRON
GYPSY
COPIA
PAO
TcMar
I
JOCKEY
LOA

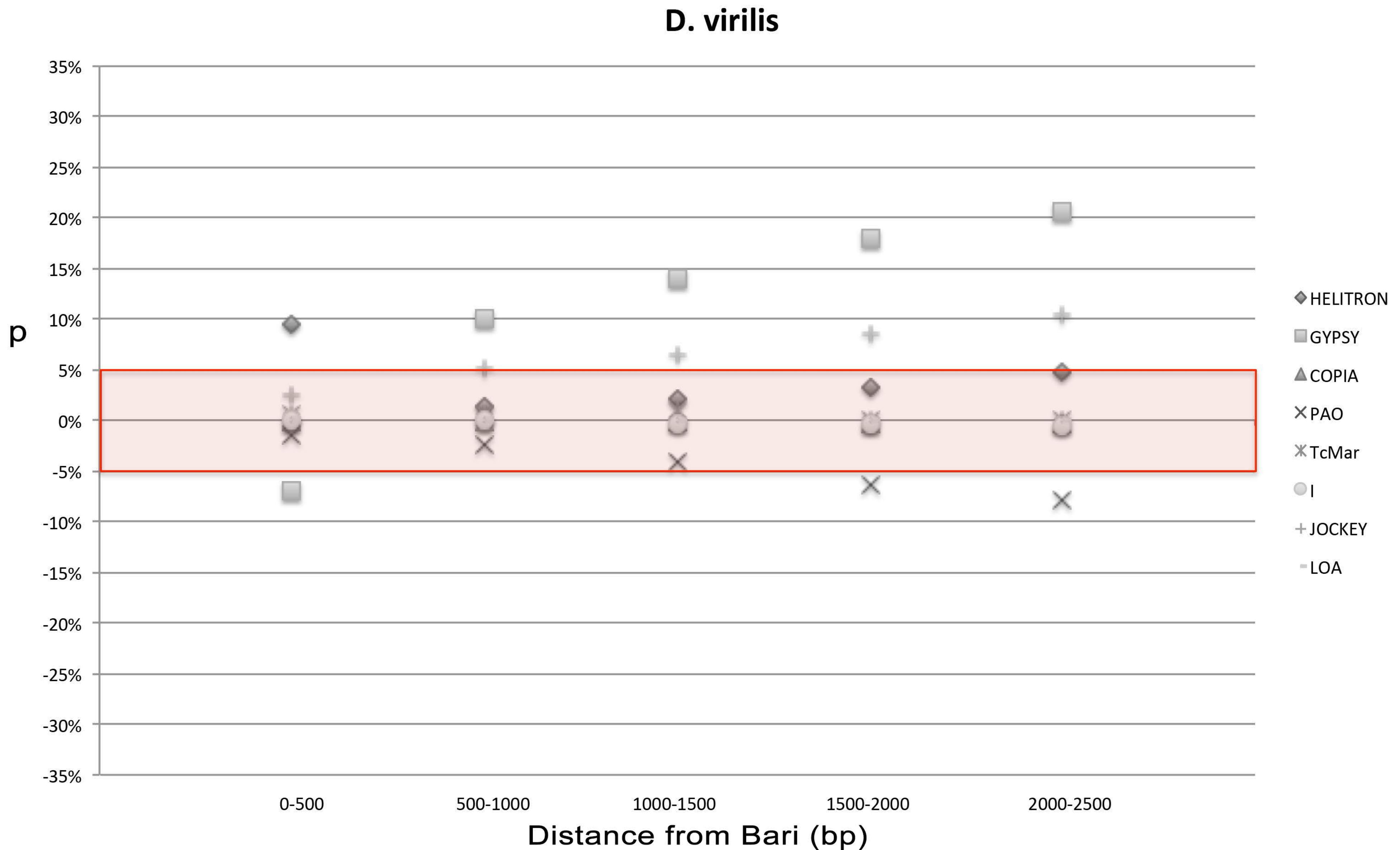

D. virilis
p
35%
30%
25%
20%
15%
10%
5%
0%
-5%
-10%
-15%
-20%
-25%
-30%
-35%
0-500
500-1000
1000-1500
1500-2000
2000-2500
Distance from Bari (bp)
HELITRON
GYPSY
COPIA
PAO
TcMar
I
JOCKEY
LOA

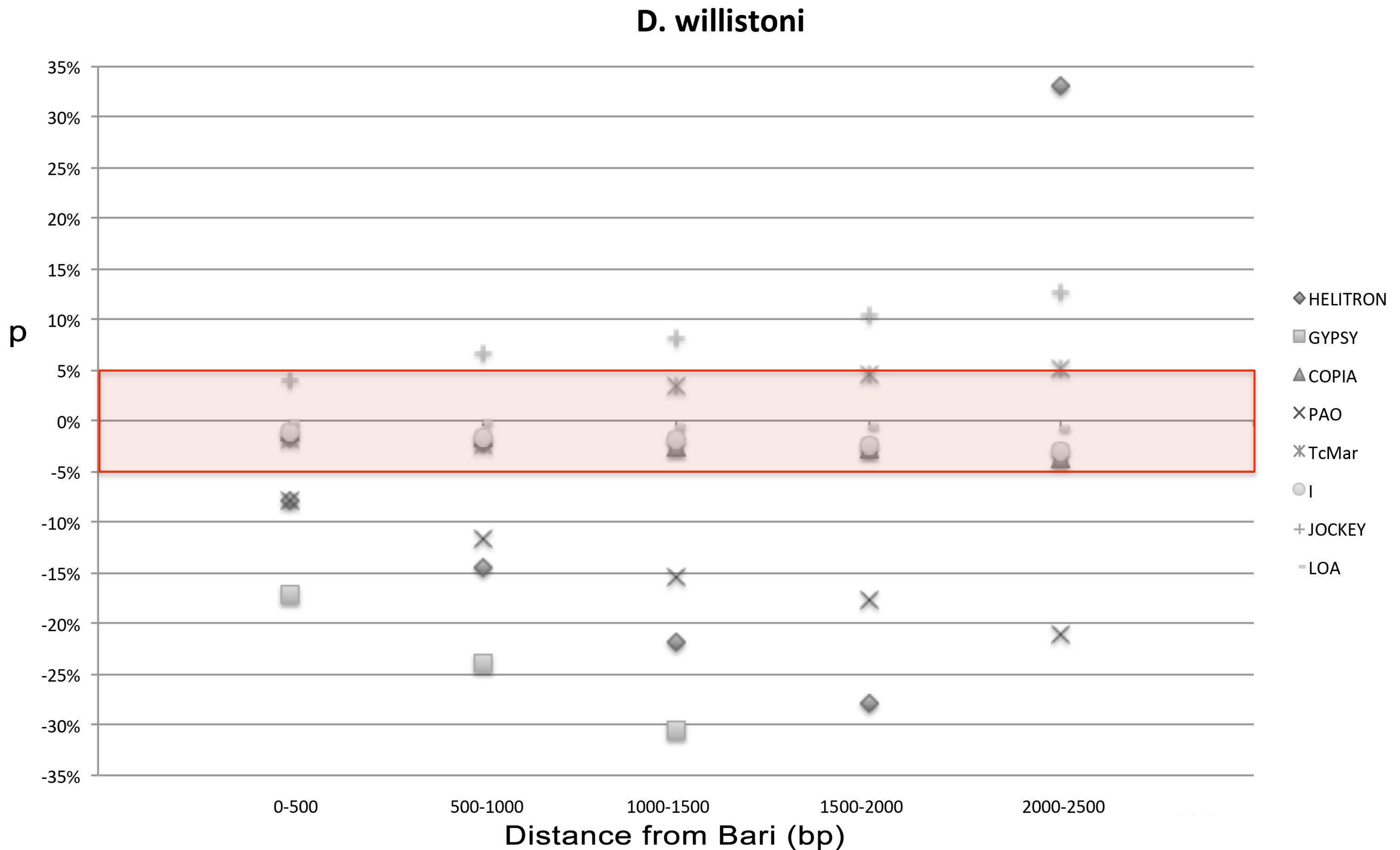
D. willistoni
35%
30%
25%
20%
15%
10%
5%
0%
-5%
-10%
-15%
-20%
-25%
-30%
-35%
p
0-500
500-1000
1000-1500
1500-2000
2000-2500
Distance from Bari (bp)
HELITRON
GYPSY
COPIA
PAO
TcMar
I
JOCKEY
LOA
