# Supplementary material for: Identification of Bari Transposons in 23 Sequenced Drosophila Genomes Reveals Novel Structural Variants, MITEs and Horizontal Transfer
Source: PLoS One. 2016 May 23;11(5):e0156014. doi: 10.1371/journal.pone.0156014 (PMC4877112; doi:10.1371/journal.pone.0156014)
Supplement: S1 Table — (PDF) [file pone.0156014.s007.pdf]

Species	Assembly	GenBank Assembly accession	Assembly status	Genomic coverage	Total sequence length	Number of contig	Number of scaffold
Drosophila albomicans	DroAlb_1.0	GCA_000298335.1	Scaffold	100.0x	253560284	188324	26354
Drosophila virilis	dvir_caf1	GCA_000005245.1	Scaffold	8.0x	206026697	18382	13530
Drosophila mojavensis	dmoj_caf1	GCA_000005175.1	Scaffold	8.2x	193826310	11874	6841
Drosophila pseudoobscura	Dpse_3.0	GCA_000001765.2	Chromosome	9.7x Sanger; 24x PacBio	152696384	6823	4790
Drosophila grimshawi	dgri_caf1	GCA_000005155.1	Scaffold	7.9x	200467819	24157	17440
Drosophila miranda	DroMir_2.2	GCA_000269505.2	Chromosome	95.0x	136728780	7031	NA
Drosophila biarmipes	Dbia_2.0	GCA_000233415.2	Scaffold	186.9x	169378599	7856	5523
Drosophila takahashii	Dtak_2.0	GCA_000224235.2	Scaffold	242.1x	182106768	9703	5733
Drosophila ficusphila	Dfic_2.0	GCA_000220665.2	Scaffold	208.7x	152439475	9152	5754
Drosophila elegans	Dele_2.0	GCA_000224195.2	Scaffold	204.9x	171267669	8403	5429
Drosophila sechellia	dsec_caf1	GCA_000005215.1	Scaffold	4.9x	166592095	21426	14731
Drosophila suzukii	Dsuzukii.v01	GCA_000472105.1	Scaffold	195x	232923092	24878	8680
Drosophila persimilis	dper_caf1	GCA_000005195.1	Scaffold	4.1x	188374079	26813	12838
Drosophila willistoni	dwil_caf1	GCA_000005925.1	Scaffold	8.4x	235516348	20358	14838
Drosophila erecta	dere_caf1	GCA_000005135.1	Scaffold	10.6x	152712140	7610	5124
Drosophila ananassae	dana_caf1	GCA_000005115.1	Scaffold	8.9x	230993012	20532	13749
Drosophila simulans	ASM75419v2	GCA_000754195.2	Chromosome	75.0x	124966452	9975	NA
Drosophila yakuba	dyak_caf1	GCA_000005975.1	Chromosome	9.1x	165709965	13441	8123
Drosophila melanogaster	Release 6 plus ISO1 MT)	GCA_000001215.4	Chromosome	NA	143726002	2422	NA
Drosophila eugracilis	Deug_2.0	GCA_000236325.2	Assembly level: Scaffold	218.1x	156942009	7568	4946
Drosophila rhopaloa	Drho_2.0	GCA_000236305.2	Assembly level: Scaffold	214.5x	197375704	34033	22819
Drosophila kikkawai	Dkik_2.0	GCA_000224215.2	Assembly level: Scaffold	181.7x	164292578	8343	5141
Drosophila bipectinata	Dbip_2.0	GCA_000236285.2	Assembly level: Scaffold	266.3x	167263958	8675	5500
