# Supplementary material for: Identification of Bari Transposons in 23 Sequenced Drosophila Genomes Reveals Novel Structural Variants, MITEs and Horizontal Transfer
Source: PLoS One. 2016 May 23;11(5):e0156014. doi: 10.1371/journal.pone.0156014 (PMC4877112; doi:10.1371/journal.pone.0156014)
Supplement: S4 Table — (PDF) [file pone.0156014.s010.pdf]

INTER-SPECIES ANALYSIS (Figure 3A)			
**Subfamily**	**Kruskal-Wallis test**		**TUKEY TEST**
**Bari1**	p=0,203227355		NA
**Bari2**	p=0,129527813		NA
**Bari3**	p=0,330090937		NA

INTRA-SPECIES ANALYSIS (Figure 3B, 3C, 3D; supplemental figure 1)				
**Species**	**Kruskal-Wallis test**		**TUKEY TEST**	
			LEFT vs CDS	* p<0.05
**D. ananassae**	p=0,00789941		LEFT vs RIGHT	** p<0.01
			CDS vs RIGHT	Not significant
			LEFT vs CDS	* p<0.05
**D. miranda**	p=0,000598		LEFT vs RIGHT	** p<0.01
			CDS vs RIGHT	Not significant
			LEFT vs CDS	* p<0.05
**D. pseudoobscura**	p=0,012510843		LEFT vs RIGHT	Not significant
			CDS vs RIGHT	Not significant
			LEFT vs CDS	** p<0.01
**D. simulans**	p=2,0873E-05		LEFT vs RIGHT	** p<0.01
			CDS vs RIGHT	* p<0.05
			LEFT vs CDS	** p<0.01
**D. willistoni**	p=0,000572994		LEFT vs RIGHT	Not significant
			CDS vs RIGHT	** p<0.01
			LEFT vs CDS	Not significant
**D. erecta**	p=3,44437E-22		LEFT vs RIGHT	** p<0.01
			CDS vs RIGHT	** p<0.01

**D. persimilis**	p=0,02361038		NA	
**D. melanogaster**	p=0,74237073		NA	
**D. mojavensis**	p=0,055702142		NA	
